# A smart nanocomposite bioactive ink for controlled siRNA delivery in calvarial mesenchymal stromal cells as a minimally invasive treatment for craniosynostosis

**DOI:** 10.1093/rb/rbaf115

**Published:** 2025-11-08

**Authors:** Martina Salvati, Federica Tiberio, Noah Giacon, Alberto Augello, Gianpiero Tamburrini, Lorena Di Pietro, Alessia Vita, Domiziano Dario Tosi, Giordano Perini, Giorgia Canini, Diego Sibilia, Valentina Palmieri, Massimiliano Papi, Ornella Parolini, Luca Massimi, Alessandro Arcovito, Wanda Lattanzi

**Affiliations:** Unità Operativa Complessa di Neurochirurgia Infantile, Fondazione Policlinico Universitario A. Gemelli IRCCS, Rome 00168, Italy; Dipartimento Scienze della Vita e Sanità Pubblica, Università Cattolica del Sacro Cuore, Rome 00168, Italy; Dipartimento Scienze della Vita e Sanità Pubblica, Università Cattolica del Sacro Cuore, Rome 00168, Italy; Fondazione Policlinico Universitario A. Gemelli, IRCCS, Rome 00168, Italy; Dipartimento di Scienze Biotecnologiche di Base, Cliniche Intensivologiche e Perioperatorie, Università Cattolica del Sacro Cuore, Rome 00168, Italy; Fondazione Policlinico Universitario A. Gemelli, IRCCS, Rome 00168, Italy; Unità Operativa Complessa di Neurochirurgia Infantile, Fondazione Policlinico Universitario A. Gemelli IRCCS, Rome 00168, Italy; Dipartimento di Neuroscienze, Università Cattolica del Sacro Cuore, Rome 00168, Italy; Dipartimento Scienze della Vita e Sanità Pubblica, Università Cattolica del Sacro Cuore, Rome 00168, Italy; Fondazione Policlinico Universitario A. Gemelli, IRCCS, Rome 00168, Italy; Unità Operativa Complessa di Neurochirurgia Infantile, Fondazione Policlinico Universitario A. Gemelli IRCCS, Rome 00168, Italy; Dipartimento Scienze della Vita e Sanità Pubblica, Università Cattolica del Sacro Cuore, Rome 00168, Italy; Unità Operativa Complessa di Neurochirurgia Infantile, Fondazione Policlinico Universitario A. Gemelli IRCCS, Rome 00168, Italy; Dipartimento Scienze della Vita e Sanità Pubblica, Università Cattolica del Sacro Cuore, Rome 00168, Italy; Fondazione Policlinico Universitario A. Gemelli, IRCCS, Rome 00168, Italy; Dipartimento di Neuroscienze, Università Cattolica del Sacro Cuore, Rome 00168, Italy; Dipartimento di Scienze Biotecnologiche di Base, Cliniche Intensivologiche e Perioperatorie, Università Cattolica del Sacro Cuore, Rome 00168, Italy; Fondazione Policlinico Universitario A. Gemelli, IRCCS, Rome 00168, Italy; Dipartimento di Neuroscienze, Università Cattolica del Sacro Cuore, Rome 00168, Italy; Istituto dei Sistemi Complessi, CNR, Rome 00185, Italy; Fondazione Policlinico Universitario A. Gemelli, IRCCS, Rome 00168, Italy; Dipartimento di Neuroscienze, Università Cattolica del Sacro Cuore, Rome 00168, Italy; Dipartimento Scienze della Vita e Sanità Pubblica, Università Cattolica del Sacro Cuore, Rome 00168, Italy; Fondazione IRCCS Casa Sollievo della Sofferenza, San Giovanni Rotondo, Foggia 71013, Italy; Unità Operativa Complessa di Neurochirurgia Infantile, Fondazione Policlinico Universitario A. Gemelli IRCCS, Rome 00168, Italy; Dipartimento di Neuroscienze, Università Cattolica del Sacro Cuore, Rome 00168, Italy; Fondazione Policlinico Universitario A. Gemelli, IRCCS, Rome 00168, Italy; Dipartimento di Scienze Biotecnologiche di Base, Cliniche Intensivologiche e Perioperatorie, Università Cattolica del Sacro Cuore, Rome 00168, Italy; Unità Operativa Complessa di Neurochirurgia Infantile, Fondazione Policlinico Universitario A. Gemelli IRCCS, Rome 00168, Italy; Dipartimento Scienze della Vita e Sanità Pubblica, Università Cattolica del Sacro Cuore, Rome 00168, Italy

**Keywords:** siRNA-mediated FGFR2 regulation, PLGA nanoparticles, GelMA hydrogel, Craniosynostosis, drug delivery

## Abstract

Craniosynostosis (CS), characterized by the premature fusion of cranial sutures, often results from aberrant activation of *Fibroblast growth factor receptor 2 (FGFR2)*, a major regulator of osteogenic differentiation in cranial mesenchyme. Despite surgical interventions, recurrence and complications remain common, underscoring the need for targeted molecular therapies. In this study, we developed a novel formulation of bioactive nanocomposite hydrogel-based ink designed for localized, sustained delivery of therapeutic small interfering RNAs (siRNAs) targeting *FGFR2*. The delivery system combines gelatin methacryloyl (GelMA), a biocompatible and photo-crosslinkable hydrogel, with poly-lactic-co-glycolic acid (PLGA) nanoparticles (NPs), creating an injectable and mouldable platform with potential for future craniofacial application. Selected siRNAs achieved up to 90% *FGFR2* mRNA knockdown and reduced downstream protein signalling activation, including pFGFR2 (60%), pERK1/2 (37%) and RUNX2 (43%) in patient-derived cells. PLGA NPs demonstrated high siRNA encapsulation efficiency, efficient cytoplasmic delivery and lysosomal escape. When embedded in GelMA and 3D-printed, the GelMA-NP system showed sustained NP retention and a controlled-release profile, maintaining functional gene silencing for up to 20 days. This multifunctional platform not only supports FGFR2 modulation in CS but also holds translational promise as a customizable scaffold for delivering other bioactive compounds, advancing paediatric cranioplasty outcomes.

## Graphical Abstract

**Figure:**
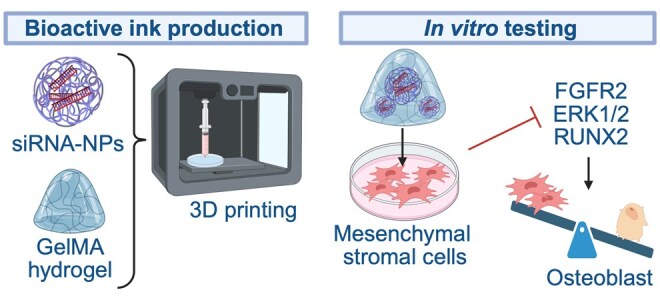

## Introduction

Craniosynostosis (CS) encompasses a wide spectrum of congenital craniofacial malformations featuring the premature ossification of one or more skull sutures, which causes a constraint in skull growth (i.e. craniostenosis) [1–3]. Current treatments rely exclusively on invasive surgical procedures, which could be multiple in complex cases, starting with early decompressive craniectomy (around 6 months of age), followed by remodelling and maxillofacial procedures [4–7]. Reoperation rates are up to 50% and increase in cases with proven single gene mutations also due to frequent post-surgical re-synostosis [8–11]. Complications, include hyperthermia, severe blood loss, wound infection, meningitis, subgalea and subcutaneous hematoma, dura mater rupture, basal encephalocele, cerebrospinal fluid leakage, intracranial empyema, venous air embolism and seizures, dramatically affecting the overall prognosis [6, 12–16]. Long-term multidisciplinary care for developmental delays or neurocognitive deficits is required in 10–20% cases despite surgical interventions [2, 13].

The urgent unmet clinical need is to reduce the frequency and invasiveness of surgical interventions, to pace down morbidities and grant sustained wellbeing and functional performance of the patients [12, 17–21]. To date, no approved pharmacological options are available to radically cure the disease by targeting the underlying developmental pathophysiology and improving the treatment outcomes. Recent advances in the genetic understanding of CS may have paved the way towards designing potential nonsurgical treatments [22]. Several studies helped identify key signalling pathways involved in premature suture fusion—primarily fibroblast growth factor receptors (FGFRs), transforming growth factor beta (TGF-β), bone morphogenic proteins (BMPs)—which are implicated in both syndromic and non-syndromic CS (NCS) forms [1, 23]. Targeting these pathways with specific drugs may offer future therapeutic alternatives. In particular, FGFR-related CS has been widely studied, and molecular strategies have been developed based on pharmacological inhibitors and RNA interference approaches [24–27]. Our group has recently demonstrated the feasibility of allele-specific silencing of *Fibroblast growth factor receptor 2* (*FGFR2)* as a suitable treatment for Crouzon syndrome [28]. This approach proved promising in modulating and normalizing the abnormal osteogenic differentiation of mesenchymal stromal cells (MSCs) in patient suture, while reducing off-target effects. However, there remains a critical challenge in achieving a sustained and prolonged delivery of therapeutic siRNAs, prior to clinical translation of such a strategy [29–31]. A suitable solution would be represented by an advanced delivery platform capable of sustained release within the calvaria niche—maintaining therapeutic action until the physiological fusion of cranial sutures. Ideally, such a system would also serve as a scaffold for cranioplasty, physically joining unfused bone structures while supporting functional bone regeneration [32, 33]. In this scenario, hydrogel-based inks may be exploited as adjuvant supports to surgery, as they easily mould and adapt to the defect shape and, with their flexibility, they can ‘glue’ and stabilize bone implants [12, 34–38]. Hydrogels have indeed attracted considerable interest in bone tissue engineering due to their excellent biocompatibility, biodegradability, low toxicity and structural similarity to the extracellular matrix (ECM) [39–42]. These features facilitate their application in treating cartilage injuries, cranial deformities and arthritis [32, 33, 35, 43]. Moreover, hydrogel-based scaffolds enable controlled drug delivery in both time and space, reducing burst release and off-target effects compared to traditional methods [39, 41, 42, 44]. Injectable hydrogels, which transition from liquid at room temperature to gel at body temperature, provide additional advantages such as minimally invasive delivery and adaptability to complex tissue geometries [12, 35–38]. Among these, gelatin methacryloyl (GelMA) stands out as a photopolymerizable hydrogel widely used for biomedical applications. GelMA combines favourable 3D structure, tuneable swelling, and porosity, allowing prolonged and localized release of therapeutic agents [45–49]. Its cell-friendly matrix, which mimics the ECM by presenting cell-attachment motifs and matrix metalloproteinase-sensitive sequences, supports cell migration, adhesion, proliferation and osteogenic differentiation, while enabling dynamic matrix remodelling [34, 45, 48]. GelMA’s ability to undergo *in situ* photo-cross-linking after injection enables defect-conforming administration, making it particularly promising for bone defect regeneration and as an adjuvant to surgical interventions [40, 50–52]. However, despite these versatile features, GelMA’s hydrophilic nature and dense cross-linked network can limit the diffusion of macromolecules such as nucleic acids—including small interfering RNA (siRNA)—thereby reducing their intracellular delivery efficiency in certain therapeutic applications [53, 54]. To overcome these limitations, GelMA-based nanocomposites have been developed by embedding various nanomaterials within the hydrogel matrix, enhancing mechanical strength, rheological behaviour, and biological functionality [32]. The resulting hybrid hydrogels provide a versatile platform that supports improved cell interactions, increased bioactivity, and more efficient, sustained cargo delivery [32, 55]. In particular, the integration of nanoparticles (NPs) with GelMA yields smart, bioactive inks with tuneable mechanical properties and targeted, controlled-release profiles [43, 48, 56]. These nanocomposite systems can encapsulate a wide range of therapeutic molecules and release them in a sustained manner, thereby extending the duration of action compared with unmodified hydrogels [48, 56–59].

Poly-lactic-co-glycolic acid (PLGA) is among the most widely exploited polymers for NP formulation. PLGA is a synthetic biopolymer regarded as safe by US Food and Drug Administration (FDA), already exploited for the delivery of the BMPs [60, 61], fibroblast growth factors (FGF) or insulin growth factor in bone tissue engineering approaches and proved suitable for enhancing bone regeneration and defects’ healing [62–66]. PLGA features tuneable properties, including a high biocompatibility and biodegradability, good solubility, variable particle size, regular morphology, large surface area, favourable pharmacokinetic property, sustained release capability and low toxicity. Furthermore, after cellular endocytosis and rapid endo-lysosomal escape, PLGA NPs are able to release their payloads to the cytoplasmic space [67, 68]. Overall, these make PLGA NPs suitable for intracellular delivery of several bioactive compounds, including siRNAs [67, 69–76].

In this study, we developed and validated *in vitro* a functionalized hydrogel-based ink embedded with PLGA NPs for controlled release of therapeutic siRNAs able to modulate the FGFR2 signalling in cells isolated from CS patients’ sutures. The designed strategy is intended for a prompt clinical translation in paediatric cranioplastic surgery, as an injectable biomaterial, able to seal bone grafts and deliver the bioactive small molecules at the implant site. This would promote a functional bone healing while impeding the aberrant ossification occurring in CS patients [12, 77]. Overall, this technology aims to improve the outcome of CS for whom complex skull remodelling interventions remain the gold standard therapeutic approach [5, 6, 15].

## Materials and methods

### Patient enrolment

This study enrolled 12 patients affected by sagittal NCS and 2 patients affected by Crouzon syndrome, an *FGFR2*-related syndromic CS, who were under care at the Paediatric Neurosurgery Unit of the Fondazione Policlinico Universitario A. Gemelli IRCCS in Rome, Italy (National reference centre for the treatment of CS within the European Reference Network for rare and/or complex craniofacial and otolaryngological disorders, ERN CRANIO https://www.ern-cranio.eu/). *FGFR2* hotspot mutations in Crouzon patients were discovered by Sanger sequence analysis [Crouzon patient#1: NM_000141.5(FGFR2):c.983A>G (p.Tyr328Cys); Crouzon patient#2: NM_000141.5(FGFR2):c.1024T>C p.(Cys342Arg)]. The experimental protocol was approved by the Ethics Committee (Protocol ID #4876 and #6830) of Università Cattolica del Sacro Cuore (Rome, Italy) and involved collecting skull suture samples as surgical tissue waste during decompressive craniectomy of CS patients.

### MSC isolation and expansion

Calvarial-derived mesenchymal stromal cells (CMSCs) were isolated in primary culture from calvaria bone tissue fragments of CS patients enrolled, according to a standardized protocol already described elsewhere and extensively characterised in earlier studies [28, 78–80]. CMSCs isolated from unfused cranial sutures were used as control cells for baseline gene and protein expression analyses (see Materials and Methods and Results sections). Once reaching confluence, primary cells were expanded until passage 7 and used as an *in vitro* model of CS disorders.

### *FGFR2* silencing assay

Given the presence of well-characterized activating mutations in the FGFR2 gene in Crouzon syndrome, and to investigate the pathological relevance of FGFR2 signalling also in non-syndromic craniosynostosis (NCS), we first assessed the basal expression levels of *FGFR2* in CMSCs derived from NCS patients compared to control CMSCs. This comparative analysis aimed to strengthen the rationale for targeting FGFR2 in NCS contexts, where no known activating mutations are present. To this purpose, CMSCs from 12 NCS patients were plated in 12-well plates at a density of 3 × 10^4^ cells per well. The day after seeding, cells were pooled in three separate pools, each consisting of CMSCs from four different patients, and analysed at both gene and protein levels (see Materials and Methods).

For the silencing assay, a pool of two commercially available siRNA molecules (Integrated DNA Technologies, IDT, Coralville, IA, USA) targeting different regions of the *FGFR2* gene open reading frame was selected and tested in patient-derived CMSC cultures (https://eu.idtdna.com/site/order/tool/index/DSIRNA_PREDESIGN, accessed on September 2023). Briefly, NCS-derived CMSCs were seeded in 6-well plates at a density of 6 × 10^4^ cells/well and treated with scalar concentration of the pool of FGFR2 siRNAs (5, 10, 25, 50 nM) using Lipofectamine RNAimax (Thermo Fisher Scientific, Inc., Waltham, MA, USA) for siRNA transfection according to manufacturer’s protocol, for 48 h. The siRNA concentration required to achieve at least a 70% mRNA knockdown was then selected to treat CMSCs derived from Crouzon patients.

Transcript and protein expression analyses were performed to assess the silencing yield of FGFR2 and of its downstream target obtained with siRNA treatment on CMSCs, as detailed below (see Materials and Methods).

To assess potential off-target effects of *FGFR2* silencing, the transcript levels of closely related FGFR family members, *Fibroblast growth factor receptor-1 and -3* (*FGFR1* and *FGFR3*), were also analysed following siRNA treatment (see Materials and Methods).

In addition, to evaluate potential rebound or compensatory mechanisms after siRNA treatment, a rescue experiment was performed. CMSCs were treated with FGFR2 siRNA for 48 h, after which the medium was replaced and cells were cultured without siRNA for an additional 24 or 96 h. *FGFR2* expression was measured at each time point (see Materials and Methods). Cells treated with Lipofectamine only (sham-transfected) and those treated with a scrambled negative control siRNA (NC siRNA, IDT) served as reference controls.

### *FGFR2* gene expression analysis

After siRNA-treatments, RNA was extracted from cell lysate using TRIzol Reagent (Zymo Research Corporation, CA, USA) according to the manufacturer’s protocol. RNA was quantified through droplet/microvolume spectrophotometer Nanodrop (NanoDrop OneC—UV-Vis Spectrophotometer, Thermo Fisher Scientific) and reverse transcribed to cDNA using PrimeScript™ RT reagent Kit (TaKaRa Bio USA, Inc) for performing gene expression analysis by real-time polymerase chain reaction (qPCR).

Target gene expression was quantified by SYBR green-based qPCR, with GoTaq^®^ qPCR Master Mix (Promega, Madison, WI, USA) using 2 µl of cDNA (5 ng/µL) from each sample. qPCR was carried out through QuantStudio™ Real-Time PCR 1 (Thermo Fisher Scientific). The primers used were purchased from Integrated DNA Technologies, IDT. The sequences used were as follows: human *FGFR2*-Forward: 5′- GGAGACAGGTAACAGTTTCGG-3′, Reverse: 5′-CCAGCGGGGTGTTGGAGTTC-3′; human *FGFR1*-Forward: 5′-TCGAGCTCACTGTGGAGTAT-3′, Reverse: 5′-CCACATCCCAGTTCTGCAGTT-3'; human *FGFR3*-Forward: 5′-ATTGGAGGCATCAAGCTGCG-3′, Reverse: 5′-AAACTTGTTCTCCACGACGC-3′; human *β-actin*-Forward: 5′-TCGTGCGTGACATTAAGGAG-3′, Reverse: 5′-CCATCTCTTGCTCGAAGTCC-3′.

The mRNA amplified signals were normalized to those of the housekeeping gene, *β-actin* and relative transcript levels were calculated using the 2^−ΔΔCt^ method [78].

### Protein expression analysis

To evaluate both FGFR2 baseline expression and the functional efficiency of siRNA-mediated knockdown, western blot analyses were performed on total protein lysates obtained from CMSCs under each experimental condition, using RIPA lysis buffer (Thermo Fisher Scientific) supplemented with a cocktail of phosphatase and protease inhibitors (Sigma-Aldrich, Saint Louis, MO, USA). Protein concentration was determined using the Pierce bicinchoninic acid (BCA) assay (Thermo Fisher Scientific). Then, 15 µg of proteins were separated on 10% SDS-polyacrylamide gels (Bio-Rad, Hercules, CA, USA) and transferred onto a nitrocellulose membrane (Bio-Rad). The membranes were blocked with 5% (w/v) non-fat dry milk in TBST (10 mM Tris pH 8.0, 150 mM NaCl, 0.2% Tween 20) at room temperature for 1 h, and then incubated overnight at 4°C with the following rabbit primary antibodies: anti-phosphorylated-FGFR2 (1:1000 in 5% BSA, Invitrogen, Thermo Fisher Scientific, #PA5-64796); anti-total form-FGFR2 (1:1000; Cell Signaling Technology, Danvers, MA, USA, #23328); anti-phosphorylated-ERK1/2 (1:1000, Cell Signaling Technology, #9101); anti-total form-ERK1/2 (1: 1000, Cell Signaling Technology, #9102); anti-RUNX2 (1: 1000, Cell Signaling Technology, #12556); anti-β-Actin (1:1000, Cell Signaling Technology, #8457). The membranes were then incubated with anti-rabbit secondary antibody (1:2000) for 1 h at room temperature. Blot images were captured using Chemidoc Image Lab analyser and software (Bio‐Rad). For each sample, band intensities of target proteins were first normalized to the corresponding β-Actin signal to account for loading variability. For phosphorylated proteins (phosphorylated-FGFR2 and phosphorylated-ERK), signal intensities were further normalized to the respective total protein (FGFR2 and ERK1/2) to assess activation status.

### Production of PLGA NPs

PLGA-based NPs were formulated with slightly different protocols to prepare empty-PLGA NPs (empty-NPs) as a control group, fluorescently labelled PLGA NPs (6-Coumarin-NPs) for trafficking analysis, and siRNA-loaded PLGA (siRNA-NPs) for delivery investigations. All PLGA (PLA:PGA ratio 50:50) NPs were prepared using the double emulsion solvent evaporation technique (w_1_/o/w_2_) as described below, using two different polymer sizes (Mw = 66 000–107 000 Da and Mw = 30 000–60 000 Da; Sigma-Aldrich), hereafter referred to as high-Mw and low-Mw, respectively. Empty-PLGA NPs were synthesized by dissolving 20 mg of PLGA in 2 mL of dichloromethane (DCM) with the addition of 200 µL of distilled water. The resultant solution was emulsified for 1 min under an ice bath using a VCX-130 *Vibra*-*Cell*™ sonicator at 45% amplitude forming a first water-in-oil (w_1_/o) emulsion. Then, a 1% polyvinyl alcohol (PVA) (Mw = 30.00–70.000; Sigma-Aldrich) solution was poured into the w_1_/o solution and sonicated for 3 min (45% amplitude), producing a water-in-oil-in-water (w_1_/o/w_2_) double emulsion. In this process, PLGA formed the polymeric shell around the inner aqueous phase [72]. 6-Coumarin-NPs were formulated using 20 mg of PLGA and 5 mg of 6-Coumarin, a fluorescent probe widely used for staining polymer particles [81], according to the protocol described above. For siRNA-NPs production, 200 µL of nuclease-free water containing 50 µM *FGFR2* siRNA pool and poly-L-lysine (PLL, 0.1% w/v; Mw = 150 000 Da; Sigma-Aldrich) in 1:1 ratio with siRNAs was diluted with 2 mL DCM containing 2 mg of PLGA. The resulting solution yielded a final siRNA concentration of 1 µM. Conjugation with poly-L-lysine as a cationic agent was used to increase the loading yield of siRNA within negatively charged PLGA nanoparticles, while also improving siRNA stability and endosomal evasion to reach the cytoplasm with the aim of degrading the target mRNA [82]. For each preparation, NPs were washed by three centrifugations at 12 000 rpm for 30 min discarding the supernatant and resuspending the NP pellet in deionized water. NPs were freeze-dried and stored at −20°C.

### Physical characterization of PLGA NPs

PLGA NPs were characterised through dynamic light scattering (DLS), nanoparticle tracking analysis (NTA) and scanning electron microscopy (SEM). Specifically, the average effective size and polydispersity index (PDI) of each NP preparation were evaluated using the Zetasizer Nano S (Malvern Instruments, Malvern, UK).

NPs were diluted with distilled water, and the measurements were carried out using the DLS mode at 25°C. The obtained results were the average of at least three analyses on the same sample. The Zeta Potential (mV) of the samples was measured through the same instrument, using a DTS1070 folded capillary cell. One hundred runs of 3 seconds were performed 3 times to obtain the average reported so far.

NTA measurements were performed with NanoSight LM10-HS system (NanoSight, Amesbury, United Kingdom). NPs were diluted 1:100 in Phosphate Buffered Saline 1X (PBS-1X, Euroclone, Italy), and then the sample was injected into the sample chamber with sterile syringes (BD Discardit II, NJ, USA) until the liquid reached the tip of the nozzle. Five recordings (60 seconds) were performed at room temperature. NTA software provided high-resolution particle size distribution profiles and concentration measurements. Dilution factors were used to calculate particle concentration. Finally, SEM analysis was employed to investigate the morphological properties of PLGA NPs. Briefly, NPs were dried overnight at room temperature directly on the SEM specimen stub, then coated with a 10-nm gold layer under vacuum. SEM images were acquired using a Zeiss Supra 25 microscope (Oberkochen, BW, Germany) at 3–5 kV.

### Efficiency of siRNA encapsulation within PLGA NPs

The encapsulation efficiency of siRNA within high-Mw and low-Mw PLGA NPs was determined following purification, lyophilization, and lysis of the NPs. After synthesis, siRNA-NPs were purified through multiple centrifugation steps to remove excess PVA and unencapsulated siRNA, followed by lyophilization for storage and analysis. To quantify the encapsulated siRNA, 20 mg of lyophilized siRNA-NPs were resuspended in 1 mL of deionized water, and 1 mL of diethylacetate was added to lyse the NPs, releasing the siRNA into the aqueous phase. The mixture was centrifuged at 12 000 rpm for 20 min at room temperature, and the aqueous phase containing the released siRNA was collected. A blank control was prepared by processing 20 mg of lyophilized unloaded PLGA NPs (without siRNA) as previously described, to account for background absorbance. Spectrophotometric analysis was performed using a NanoDrop™ One/OneC Microvolume UV-Vis Spectrophotometer, with absorbance measurements taken at 260 nm to quantify nucleic acid content. The encapsulation efficiency was calculated using the equation below, where the released siRNA concentration (ng/µl) represents the siRNA recovered from lysed NPs, and the initial siRNA concentration reflects the theoretical siRNA loading from the input solution used in NP formulation.


Encapsulation efficiency (%)=Released siRNA concentration (ng/μl)*Initial siRNA concentration (ng/μl)**×100 (%) 

*Calculated based on the spectrophotometer measurement at 260 nm of lysed siRNA-NP solution.

**Calculated based on the spectrophotometer measurement at 260 nm of the solution containing the total amount of siRNA used for NP production.

This indirect lysis method successfully quantified siRNA encapsulation within the PLGA matrix while offering several methodological advantages, including accurate background correction through the use of blank controls that eliminate interference from polymer-degradation products, complete siRNA recovery through organic solvent-mediated matrix disruption, and minimized degradation artefacts by employing indirect quantification methods that reduce potential underestimation from siRNA degradation during processing. The protocol aligns with established methodologies for nucleic acid encapsulation assessment and provides reliable quantification of siRNA loading efficiency in polymeric delivery systems [83].

### Biocompatibility

To evaluate the potential cytotoxic effects of NPs, CMSCs of NCS patients were incubated with PLGA NPs, and biocompatibility was assessed using the MTT (3-(4,5-dimethylthiazol-2-yl)-2,5-diphenyltetrazolium bromide) viability assay and live-cell imaging (Incucyte^®^ Live Cell Analysis System, Sartorius^TM^). Regarding the MTT assay, which measures cell metabolic activity, CMSCs from NCS patients were seeded in 96-well plates at a density of 5 × 10^3^ cells/well in standard growth medium. The following day, cells were treated with scalar concentrations of PLGA NPs (0.02, 0.04, 0.2, 0.5 and 2.5 mg/mL). After 48 h of incubation, MTT reagent (10% v/v) was added to each well, and cells were incubated at 37°C for 4 h. Absorbance was measured at a wavelength of 570 nm using a spectrophotometer (Cytation 3 cell imaging multi-mode reader, Biotek, Winooski, VT, USA) to compare the metabolic activity of NP-treated cells with untreated controls. For the proliferation analysis, CMSCs were seeded in 24-well plates at a density of 1.5 × 10^4^ cells/well and incubated with scalar concentrations of PLGA NPs (as described above) within Incucyte system. For each condition, 9 images/well (10× objective) were acquired every 3 h until 48 h of incubation. Cell proliferation was calculated in terms of relative confluence for each well by Incucyte Basic Software.

### Cellular uptake and trafficking of PLGA NPs

6-Coumarin-NPs were used to investigate the uptake and intracellular trafficking of NPs in CMSC culture isolated from NCS patients. To preliminarily assess the dynamics of NP-cell interactions, CMSCs were seeded in 24-well plates at a density of 1.5 × 10^4^ cells/well and then treated with scalar concentrations of 6-Coumarin-NPs (0.02, 0.04, 0.2 and 1 mg/mL). Treated cells were incubated within Incucyte system, allowing real-time detection of green fluorescence (300-ms exposure) signals from fluorescently labelled NPs. Nine images per well were taken every 3 h for 24 h using a 10× objective. Fluorescence analysis was performed using the Incucyte Basic Software, which detects green objects per total area (µm^2^) for each image captured during CMSCs incubation with NPs.

To further investigate the spatial distribution of 6-Coumarin-NPs and evaluate their potential uptake within CMSCs, confocal microscopy analysis was performed. CMSCs were seeded in an 8-well chamber slide (µ-Slide 8 Well, Ibidi, Munich, Germany) at a density of 8 × 10^3^ cells/well for 24 h and then were treated with increasing concentrations of 6-Coumarin-NPs (as described above). After 24 h, CMSCs were fixed, stained using nuclear DAPI (5 µg/ml) and actin cytoskeleton staining (1:200, β-actin; Cell signaling) and analysed by confocal microscopy Nikon A1 MP+, using 60× and 100× objectives.

### Intracellular fate of PLGA NPs

To investigate the intracellular localization and lysosomal trafficking of PLGA NPs, CMSCs seeded in an 8-well chamber slide (Ibidi) as previously described, were incubated with Lysotracker Deep Red staining (Thermo Fisher Scientific) for 1 h, according to the manufacturer’s protocol. Afterwards, cells were treated with scalar concentrations of 6-Coumarin-NPs. After 3 h, confocal microscope images of treated and untreated cells (used as a control) were captured using a 100× objective on a Nikon A1 MP+ microscope. To quantify lysosomal escape, co-localization analysis between 6-Coumarin-NP fluorescence and Lysotracker Deep Red signal was performed using the ImageJ software on images acquired. The percentage of NP-associated fluorescence found outside the lysosomal compartment (non-overlapping signal) was calculated to assess the cytoplasmic distribution. To more clearly appreciate the co-localization, additional images were acquired at 2× digital zoom (total magnification 200×).

### CMSC treatment with *FGFR2* siRNA-NPs

To evaluate the suitability of PLGA NPs as an intracellular nanocarrier for selected siRNA, siRNA-NPs were tested in CMSC culture of NCS patients. Briefly, CMSCs were seeded in 6-well plates at a density of 6 × 10^4^ cells/well and treated with 2.5 mg/mL of NPs encapsulating 0.8 µM *FGFR2* siRNA. siRNA silencing efficiency was evaluated after 24, 48, 72 and 96 h of CMSC treatment with siRNA-NPs by studying *FGFR2* transcript level through gene expression analysis (as previously described in Materials and methods section titled “FGFR2 gene expression analysis”). CMSCs treated with empty-NPs were used as controls to account for any effects due to the NP carrier itself.

### Nanocomposite bioactive ink 3D printing

GelMA-based hydrogel, named GelXA, was purchased from CELLINK (USA) as ready-made syringes. GelXA is a semi-translucent hydrogel based on GelMA, alginate and xanthan gum that improves printability, with an adjustable degree of methylation of 45–55% by cross-linking agents. To enhance and optimize the local and controlled delivery strategy, a nanocomposite bioactive ink consisting of PLGA NPs combined within a GelMA hydrogel-based matrix was produced. Specifically, empty-NPs, 6-Coumarin-NPs or siRNA-NPs were mixed with GelXA, resulting in control, fluorescent or gene knockdown nanocomposite inks, respectively. The inks were sterile printed via BioX 3D bio-printer (CELLINK) in 12-well plates with a grid structure using the extrusion-based technique at a printhead temperature of 37°C and a printbed temperature of 27°C. Using a 22G nozzle, the bioactive ink was printed with an extrusion pressure of 9 kPa (300 ms preflow) and a printing speed of 10 mm/s, achieving a layer height of 0.3 mm. After printing, the NP–GelXA ink was cross-linked for 45 seconds at a wavelength of 405 nm (UV).

### NP release profile from nanocomposite bioactive ink

To identify the optimal NP concentration to be printed within the ink for subsequent release and functional studies, 6-Coumarin–NP–GelXA constructs were fabricated with a grid-like structure using scalar concentrations of NPs (2.5, 0.5, 0.05 mg/mL). Then, CMSCs derived from NCS patients were seeded (3 × 10^4^ cells/well) upon the 3D-printed inks, and live fluorescence imaging was performed over 72 h using the Incucyte Live-cell analysis system to monitor both NP retention within the hydrogel and intracellular uptake. Specifically, fluorescent signals derived from 6-Coumarin-NPs were detected by the Incucyte software as green objects/well on the 36 images/well taken every 3 h. These analyses served as a guide in selecting the most appropriate NP concentration capable of producing the best prolonged NP retention profile for downstream experiments. 3D GelXA without NPs was used as a control.

To thoroughly characterize the release profile of NPs from the GelXA matrix, 2.5 mg/mL of NPs were embedded within the GelXA printed in a grid-like structure within 12-well plates and incubated in PBS at 37°C for up to 30 days. At daily intervals, aliquots of PBS were collected from each well and replaced with fresh buffer to preserve ionic equilibrium throughout the experiment. The collected samples were analysed by NTA as described above (see Materials and Methods section) to quantify the cumulative release of NPs over time and to evaluate the size distribution of the particles released from the ink. This combined approach enabled a comprehensive evaluation of both the release kinetics and the physical stability of the NPs within the ink matrix, providing essential insights for optimizing the bioactive ink formulation for sustained therapeutic delivery.

### Biocompatibility and inflammatory response assessment of NP–GelXA ink

To gain insight into the biological effects of the NP–GelXA ink, CMSCs derived from NCS patients were seeded (3 × 10^4^ cells/well) upon the printed ink and cell proliferation was monitored over time for 72 h using live-cell imaging (Incucyte). Additionally, cell viability was quantified at 48 h after treatment with NP–GelXA ink using CellTiter-Glo luminescent assay (Promega, Madison, WI, USA), which quantifies cellular ATP levels as an indicator of metabolically active and viable cells, according to the manufacturer’s protocol. Briefly, the medium was removed from the well and replaced with fresh growth medium containing CellTiter-Glo reagent in 1:1 ratio. Fluorescence intensity was recorded with Cytation 3 reader by exciting at 550 nm and reading the emission at 600 nm. CMSCs’ growth in standard proliferation medium was used as controls.

To evaluate the immunological response elicited by the nanocomposite ink, cytokine secretion profiles were assessed. Peripheral blood mononuclear cells (PBMCs) isolated from four healthy donors were seeded at a density of 1.5 × 10^6^ cells/well onto NP–GelXA constructs and incubated for 24 h. After incubation, the cell culture supernatants were collected and centrifuged at 1400 rpm for 10 min to remove cellular debris. The levels of pro- and anti-inflammatory cytokines—including TNF-α, IL-6, IL-1β, CXCL10 (IP-10), IL-13, IL-4, IL-10, and IL-1RA—were then quantified using the Luminex Performance Human XL Cytokine Panel (Luminex Report Service, Milan, Italy).

In parallel, PBMC viability was measured from the cell pellets using the CellTiter-Glo assay to confirm that any observed changes in cytokine levels were not due to cytotoxic effects.

### *In vitro* validation of the nanocomposite bioactive ink

To validate the nanocomposite bioactive ink for the local and sustained delivery of siRNA, siRNA-NPs were produced as described before and embedded within the GelXA matrix and 3D printed within a well. The NP concentration was calculated to ensure a final siRNA content of 0.8 µM per construct. NCS patients’ CMSCs were seeded upon the 3D-printed siRNA–NP–GelXA ink for 2, 4, 14 and 20 days. At each time point, *FGFR2* gene expression was evaluated by qPCR. Untreated CMSCs and CMSCs incubated with control ink (NP–GelXA) without siRNA were used as controls.

### Statistical analysis

Data were analysed using GraphPad Prism software version 10.0. Results are presented as mean ± standard deviation (SD). Statistical differences between groups were analysed using the unpaired Student’s *t*-test (two-tailed) or one-way analysis of variance followed by Tukey’s *post hoc* test. The level of significance was set at *P *< 0.05.

## Results

### siRNA targeting *FGFR2* significantly modulates the FGFR2-related osteogenic cascade in CS patients’ cells

**Figure 1:**
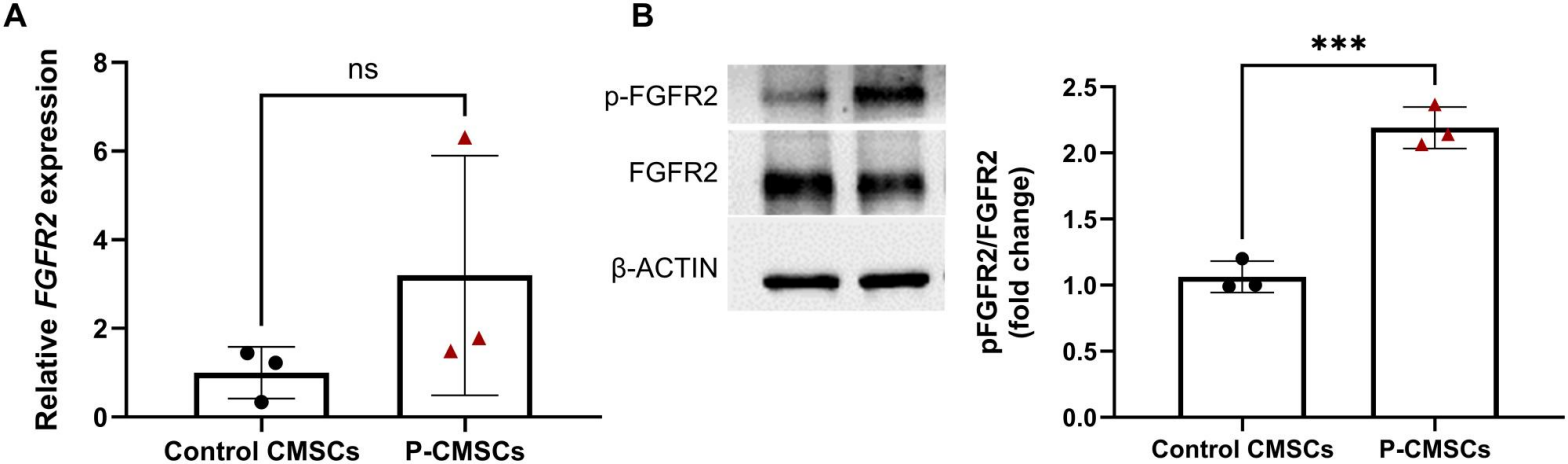
FGFR2 Baseline expression in cranial suture-derived cells. (**A**) Gene expression analysis of *FGFR2* mRNA of calvarial mesenchymal stromal cells derived from fused cranial sutures of NCS patients (named pathological calvarial mesenchymal stromal cells, P-CMSCs) compared to control cells isolated from unfused cranial bone tissue (control CMSCs). (**B**) Representative Western blot images and relative densitometric analysis of phospho-FGFR2 (pFGFR2) to total FGFR2 ratio in CMSCs from NCS patients (P-CMSCs) compared to control cells. Western blot quantifications were performed normalizing the intensity of each protein band to its corresponding β-actin signal. Data are represented as mean ± SEM from three independent cell pool (each consisting of CMSCs derived from four NCS patients, total *n* = 12). Results are shown as mean (*n* = 4) with SD (error bars). Data were analysed using the unpaired Student’s *t*-test. ***P ≤ 0.001. ns: not significant.

To explore the potential therapeutic application of a gene knockdown approach to inhibit aberrant ossification in CS, we selected the *FGFR2* gene as a suitable target. The tyrosine kinase receptor FGFR2 plays a key role in the aberrant differentiation of osteoprogenitor cells in the cranial suture mesenchyme, leading to premature ossification in both syndromic and NCS CS patients [1, 23, 84]. To support the rationale for targeting *FGFR2* in CS, we first verified whether CMSCs derived from patients with NCS exhibited abnormal basal FGFR2 activity compared to cells isolated from unfused control sutures. Gene expression analysis revealed a slight, albeit non-significant, upregulation of *FGFR2* transcripts in CMSCs derived from NCS compared to control cells (Figure 1A). This transcriptional alteration was accompanied by increased activation of the phosphorylated form of FGFR2 at the protein level, as demonstrated by western blot analysis showing a higher phospho-FGFR2/total FGFR2 ratio in NCS cells compared to controls (Figure 1B), supporting a pathological overactivation of this signalling axis even in the absence of diagnosed mutations.

**Figure 2:**
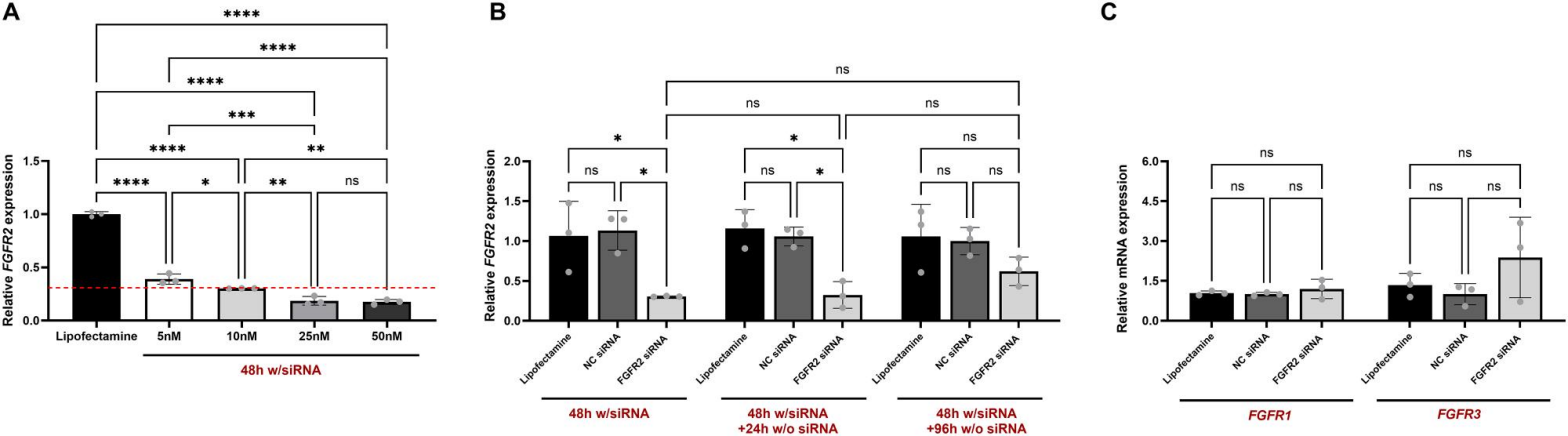
siRNA selection and validation for *FGFR2* silencing in CMSCs. (**A**) *FGFR2* expression analysis in NCS-patient-derived cells treated with scalar concentration (5, 10, 25, 50 nM) of a siRNA pool targeting *FGFR2* delivered by Lipofectamine for 48 h. Cells treated with Lipofectamine alone were used as vehicle controls (Lipofectamine). Red dashed line: gene inhibition threshold (70%). (**B**) Gene expression analysis of *FGFR2* in CMSCs treated with 10 nM FGFR2-targeting siRNAs for 48 h followed by siRNA removal and continued culture in standard medium for an additional 24 or 96 h. Cells treated with scrambled negative control siRNA (NC) and Lipofectamine were used as controls. (**C**) qPCR analysis performed on CMSCs 48 h after transfection with 10 nM FGFR2-targeting siRNAs to assess potential off-target effects. Transcript levels of *FGFR1* and *FGFR3* were measured and normalized to *β-actin*. Cells treated with NC and Lipofectamine were used as controls. (**A–C**) Transcript levels were measured by qPCR and normalized to *β-actin*. Each condition has been performed in biological triplicates (*n* = 3). Results are shown as mean (*n* = 4) with SD (error bars). Data were analysed using the one-way ANOVA. *P ≤ 0.05, **P ≤ 0.01, ***P ≤ 0.001, ****P ≤ 0.0001. ns: not significant.

Based on these findings, we proceeded to evaluate a gene knockdown strategy for slowing down *FGFR2* expression. To achieve this, a pool of siRNAs targeting *FGFR2* was used to downregulate the disease-associated overactive FGFR2 intracellular signalling in CMSCs. NCS cells were treated with scalar concentrations of siRNAs transfected via Lipofectamine to identify the most effective concentration for *in vitro* use. In this setup, after 48 h of treatment, a statistically significant downregulation of *FGFR2* transcript levels was observed in CMSCs, showing a dose-dependent trend of response, starting from the minimal siRNA dose of 5 nM (Figure 2A). Specifically, treatment with 5, 10, 25 and 50 nM of siRNAs resulted in 60%, 70%, 80% and 85% *FGFR2* inhibition, respectively, compared to Lipofectamine-only controls (Figure 2A).

Subsequent *in vitro* analyses were carried out using 10 nM siRNAs, identified as the lowest effective concentration to achieve at least 70% gene target reduction (dashed red line in Figure 2A), considered as a threshold for active siRNAs to achieve a relevant clinical impact [85].

We then investigated the temporal stability of the siRNA-mediated silencing effect and possible compensatory gene expression. After 48 h of siRNA treatment, the siRNA-containing medium was removed, and CMSCs were cultured for an additional 24 or 96 h under standard conditions. Gene expression analysis showed that FGFR2 mRNA remained strongly downregulated 24 h after treatment withdrawal (∼65% reduction compared to controls), with only a slight decrease from the ∼70% knockdown initially observed. At 96 h, FGFR2 levels partially recovered, with inhibition decreasing to ∼39%; however, this change was not statistically significant when compared to controls (Figure 2B). To further investigate the specificity of *FGFR2* silencing, we evaluated potential off-target effects by measuring the expression levels of *FGFR1* and *FGFR3*, two closely related members of the FGFR family. In particular, qPCR analysis showed no significant changes in the expression of either gene after 48 h of treatment with *FGFR2*-targeted siRNA (Figure 2C), confirming the selectivity of the siRNA pool used.

These results confirmed the transient and reversible nature of siRNA-mediated knockdown, with no evidence of compensatory overexpression or off-target effects in response to gene silencing. In addition, no significant differences were observed between cells treated with Lipofectamine-only and those treated with scrambled negative control siRNA (NC), confirming that neither vehicle nor non-specific siRNA affected *FGFR2* expression or induced off-target effects (Figure 2B and C). These findings validated the use of Lipofectamine-only control for the remaining assays.

**Figure 3:**
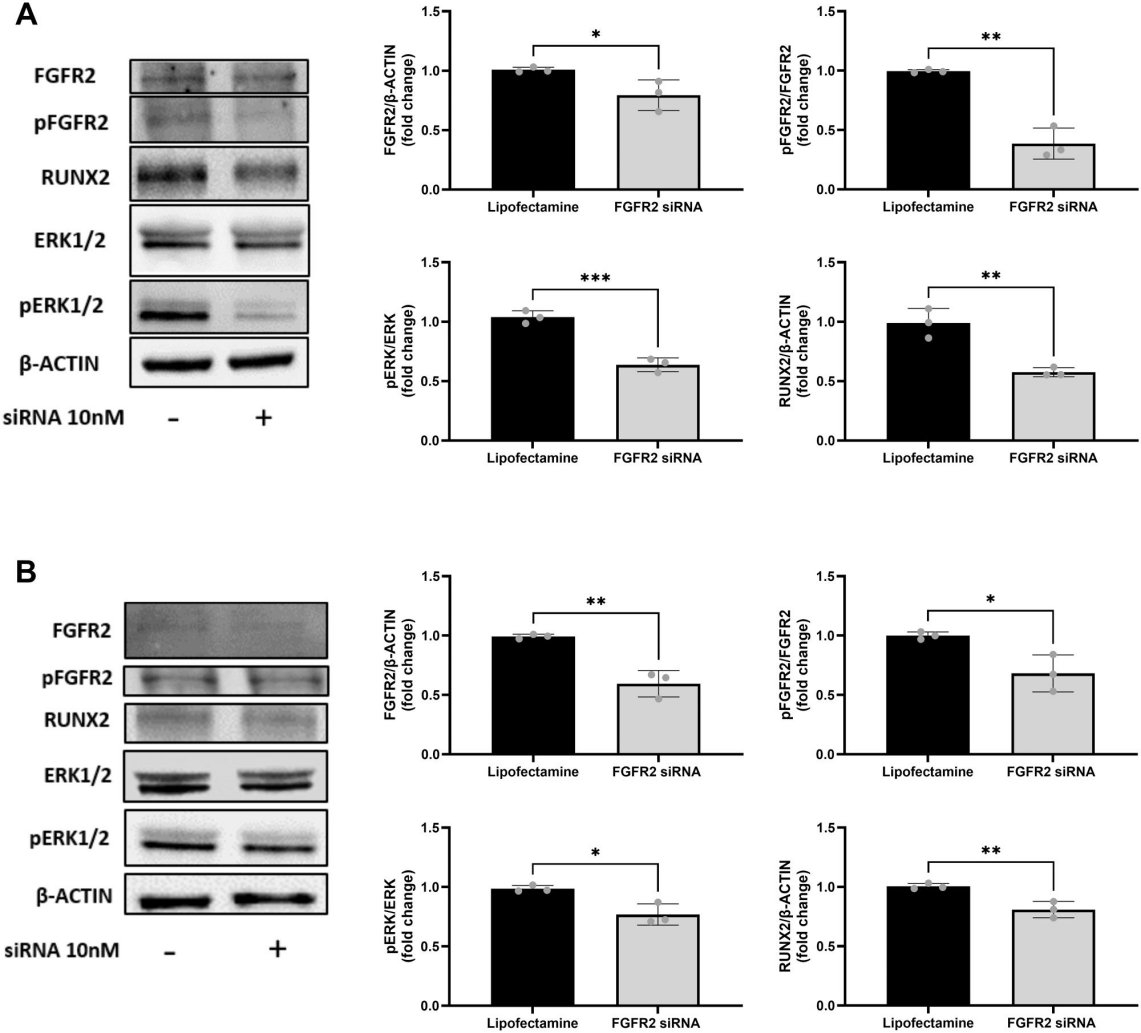
FGFR2-siRNA silencing yield in patient-derived cells. (**A**) Representative Western blot images and relative densitometric analysis of total FGFR2 (FGFR2), phospho-FGFR2 (pFGFR2), phospho-ERK1/2 (pERK) and RUNX2 after 48 h of siRNA treatment (10 nM) in NCS-derived CMSCs. (**B**) Representative Western blot images and relative densitometric analysis of total FGFR2 (FGFR2), phospho-FGFR2 (pFGFR2), phospho-ERK1/2 (pERK) and RUNX2 after 48 h of Crouzon patient-derived CMSCs treatment with siRNAs (10 nM). (**A and B**) Band intensities were normalized to β-actin and to their respective total proteins for phosphorylated forms. Data are representative of three independent experiments (*n* = 3) and were analysed using the unpaired Student’s *t*-test. *P ≤ 0.05, **P ≤ 0.01, ***P ≤ 0.001.

At the protein level, siRNA treatment resulted in a 20% reduction in total FGFR2 expression in NCS-derived cells compared to vehicle controls, as assessed by densitometric analysis normalized to β-Actin (Figure 3A). Phosphorylated FGFR2 (pFGFR2) levels, normalized to total FGFR2, were reduced by 60%, indicating a marked decrease in receptor activation in NCS. Consistently, downstream effectors of FGFR2 signalling, including phosphorylated-ERK (pERK, normalized to total ERK) and RUNX2, were decreased by 37% and 43%, respectively, relative to controls (Figure 3A). These results highlighted the effective FGFR2 knockdown at the protein level and the subsequent downregulation of its signalling pathway.

Thereafter, siRNA efficacy was also assessed in CMSCs isolated from 2 Crouzon patients (see Materials and Methods section) carrying recognized pathogenic mutations in *FGFR2* gene. Gene expression analysis demonstrated that 48 h of siRNA treatment inhibited FGFR2 mRNA levels by 90% in Crouzon cells compared to untreated controls (Supplementary Figure S1). Western blot analysis confirmed that FGFR2 receptor levels were downregulated by 40% in treated cells compared to Lipofectamine-treated controls (Figure 3B). Additionally, pFGFR2/FGFR2 ratio was reduced by 30% following siRNA treatment. This reduction resulted in a 20% decrease in the activation of downstream effectors such as pERK and RUNX2, indicating a functional downregulation of the FGFR2 signalling cascade (Figure 3B). Overall, these data confirmed that the siRNAs treatment decreased *FGFR2* gene expression and, consequently, reduced protein availability and functionality.

### PLGA NPs are suitable biocompatible nanocarriers for siRNAs delivery in CMSCs

In order to develop and validate highly biocompatible nanocarriers for the delivery of the selected therapeutic siRNAs, we efficiently produced empty-NPs, 6-Coumarin-NPs and siRNA-NPs using two different molecular weight PLGA polymers to evaluate the impact of polymer characteristics on NP properties.

For NPs prepared with high-Mw PLGA, comprehensive characterization was performed using multiple analytical techniques. DLS measurements revealed particle sizes of 213 ± 3 nm in diameter for empty-NPs, 247 ± 3 nm for 6-Coumarin-NPs and 297 ± 7.5 nm for siRNA-NPs (Supplementary Figure S2A–D). The variation in particle size corresponded to the different types and amounts of cargo encapsulated within the PLGA matrix. The PDI values were 0.08 for empty-NPs, 0.2 for siRNA-NPs, and 0.31 for 6-Coumarin-NPs, demonstrating acceptable size distributions with the empty particles showing the highest uniformity.

**Figure 4:**
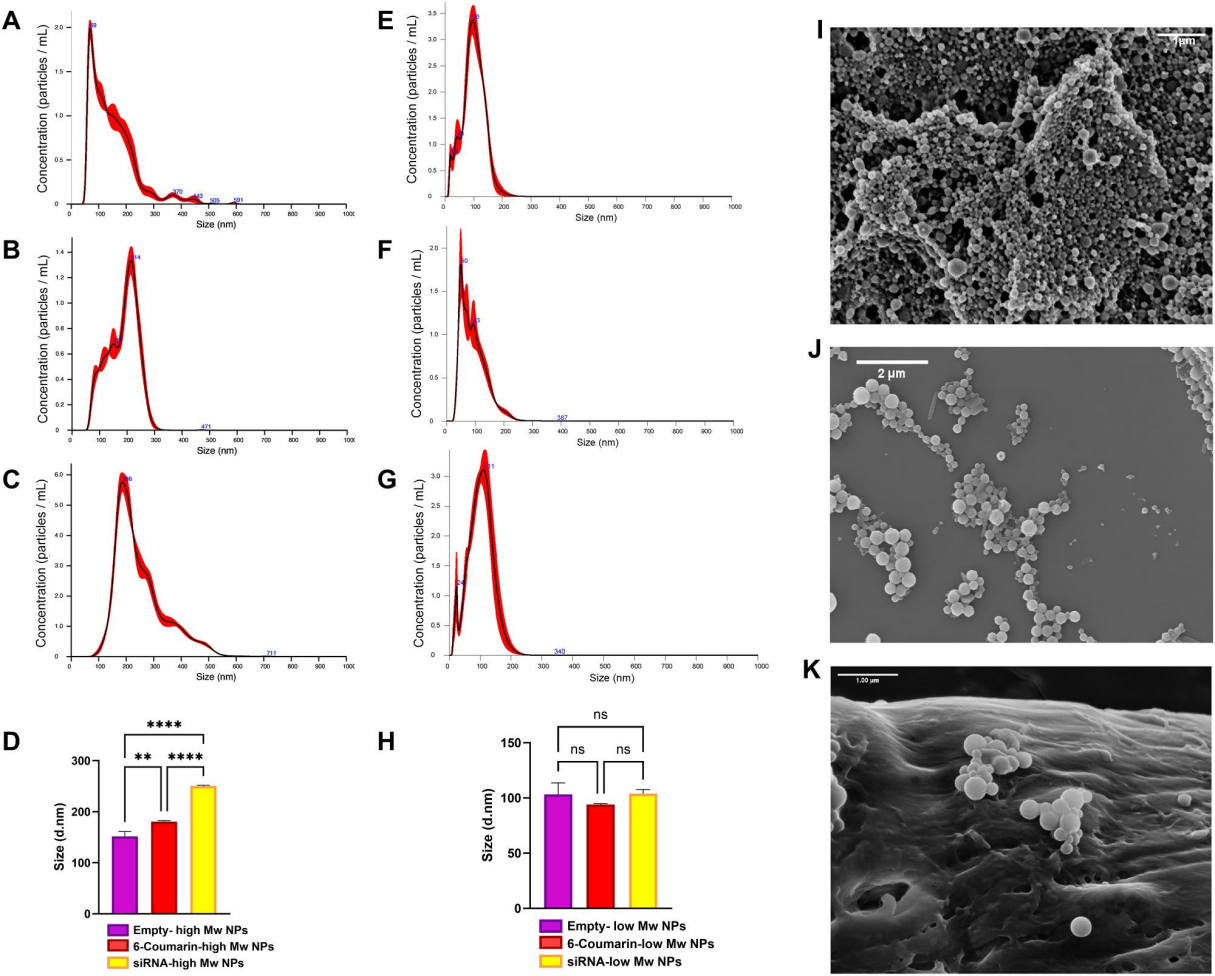
PLGA NPs characterization. (**A**–**C**) Nanoparticle tracking analysis (NTA) of high-Mw PLGA NPs: (**A**) empty-NPs, (**B**) 6-Coumarin-loaded NPs, and (**C**) siRNA-loaded NPs, showing size distribution profiles. (**D**) Summary bar graph comparing the mean particle diameters of high-Mw PLGA NPs across the three formulations. Statistical analysis was performed using one-way ANOVA. **P ≤ 0.01, ****P ≤ 0.0001. (**E–G**) NTA profiles of low-Mw PLGA NPs: (**E**) empty-NPs, (**F**) 6-Coumarin-loaded NPs and (**G**) siRNA-loaded NPs. (**H**) Summary bar graph comparing mean particle diameters of low-Mw PLGA NPs across the three formulations. Statistical analysis was performed using one-way ANOVA. ns: not significant. (**I–K**) Representative scanning electron microscopy (SEM) images of low-Mw PLGA NPs: (**I**) empty, (**J**) 6-Coumarin-loaded and (**K**) siRNA-loaded. **P ≤ 0.01, ****P ≤ 0.0001. ns: not significant.

Particle concentration was calculated using nanodrop software, accounting for dilution factors, which returned a consistent value of 1 × 10^10^ particles/mL for all formulations. The Z-potential measurements showed values of -5.47 ± 0.1 mV for empty-NPs and −1.51 ± 0.3 mV for siRNA-NPs (Supplementary Figure S2E), indicating slightly negative surface charges suitable for biological applications. NTA technology was also employed to cross-validate DLS results and provided additional size distribution information for both polymer formulations. For high-Mw PLGA NPs, NTA analyses showed a particle size distribution ranging from 100 to 300 nm, with mean diameters of 149.3 ± 2.6 nm for empty-NPs, 183.7 ± 2.7 nm for 6-Coumarin-NPs and 250.2 ± 1.2 nm for siRNA-NPs (Figure 4A–D). By contrast, when low-Mw PLGA was used for NP preparation, NTA measurements revealed significantly smaller and more uniform particle sizes across all formulations: empty-NPs measured 100 ± 4.6 nm, 6-Coumarin-NPs measured 93.2 ± 1.5 nm and siRNA-NPs measured 99.7 ± 7 nm in diameter (Figure 4E–H). These results highlighted that the molecular weight of the PLGA polymer influences the final NP size, with lower molecular weight polymers yielding more compact nanostructures regardless of the encapsulated cargo type.

The encapsulation efficiency was determined by comparing the absorbance at 260 nm, as described in the Materials and Methods section, and ranged from 40% to 82% for low- and high-Mw PLGA NPs, respectively. Given the higher homogeneity of the low-Mw PLGA, as demonstrated by NTA, we then assessed its morphology using SEM. This revealed a homogeneous spherical morphology with smooth surfaces across all formulations (Figure 4I–K). The particles appeared uniform in shape regardless of cargo type, indicating that the loading process did not significantly affect their structural characteristics.

**Figure 5:**
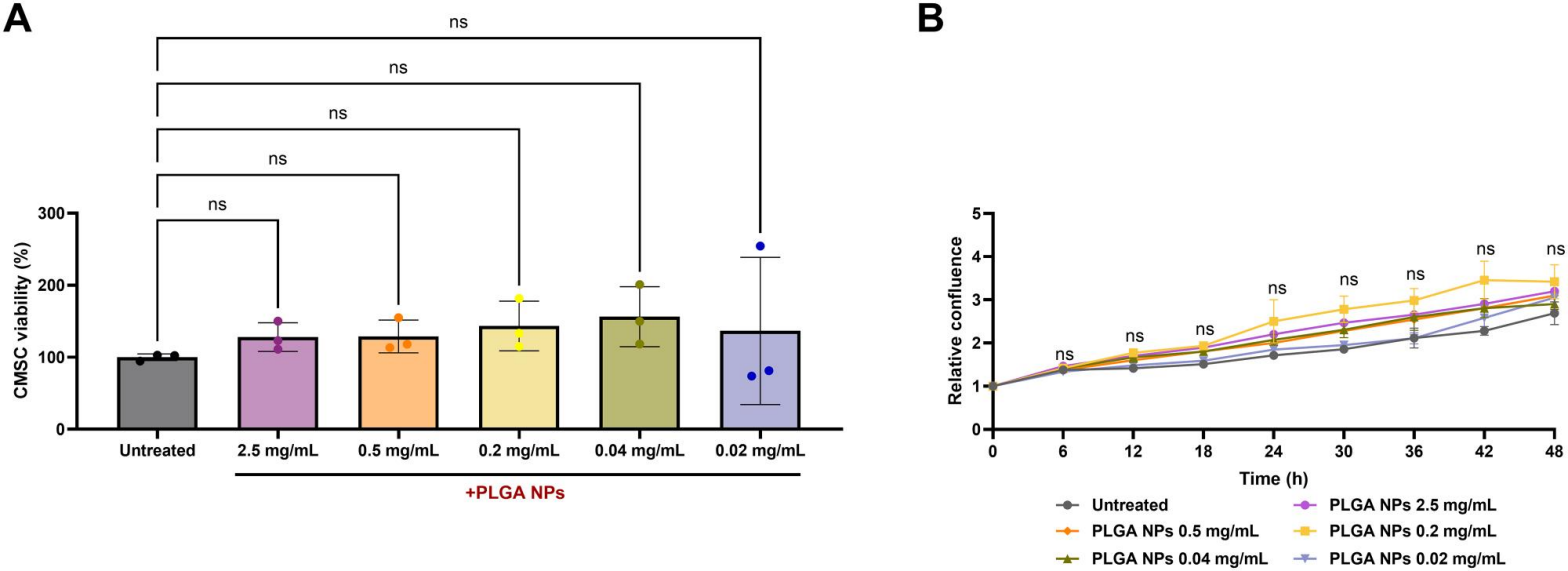
Biocompatibility evaluation of PLGA NPs. (**A**) MTT viability assay after CMSCs incubation with scalar concentrations of NPs (2.5, 0.5, 0.2, 0.04, 0.02 mg/mL) for 48 h. Cytotoxicity data were normalized to untreated controls. (**B**) Proliferation analysis of CMSCs incubated for 48 h with PLGA NPs (2.5, 0.5, 0.2, 0.04, 0.02 mg/mL) by live-cell imaging analysis (Incucyte system). Untreated CMSCs were used as controls. Data were analysed using the one-way ANOVA. All data are presented as mean (*n* = 3) with SD. ns: not significant.

Biocompatibility studies were conducted using the high-Mw PLGA formulation, given the comparable safety profile of nanoparticles sized 100–300 nm, unlike smaller ones (10–30 nm), which exhibit greater cytotoxicity [86]. In this regard, the MTT viability assay was assessed to analyse the metabolic activity of CMSCs cultured with NPs (Figure 5A), and cell confluence was investigated to study the impact of the NPs on CMSC proliferation (Figure 5B). The MTT assay showed no significant differences in the viability of cells treated with PLGA NPs for 48 h at all tested concentrations (0.02, 0.04, 0.2, 0.5, 2.5 mg/mL) compared with the untreated controls, indicating that PLGA NPs did not affect cellular metabolism (Figure 5A). The proliferation assay allowed studying cell growth in real time during the treatment with scalar concentrations of PLGA NPs (0.02, 0.04, 0.2, 0.5 and 2.5 mg/mL) for 48 h. Although CMSC proliferation showed a dose-dependent increase over 48 h of treatment with PLGA, no statistically significant differences in cell confluence were observed compared to the controls (Figure 5B). Altogether, the two assays confirmed the high biocompatibility of the produced PLGA NPs within the tested cellular model.

### PLGA NPs are efficiently internalized by CMSCs, distributing within their cytoplasm

**Figure 6:**
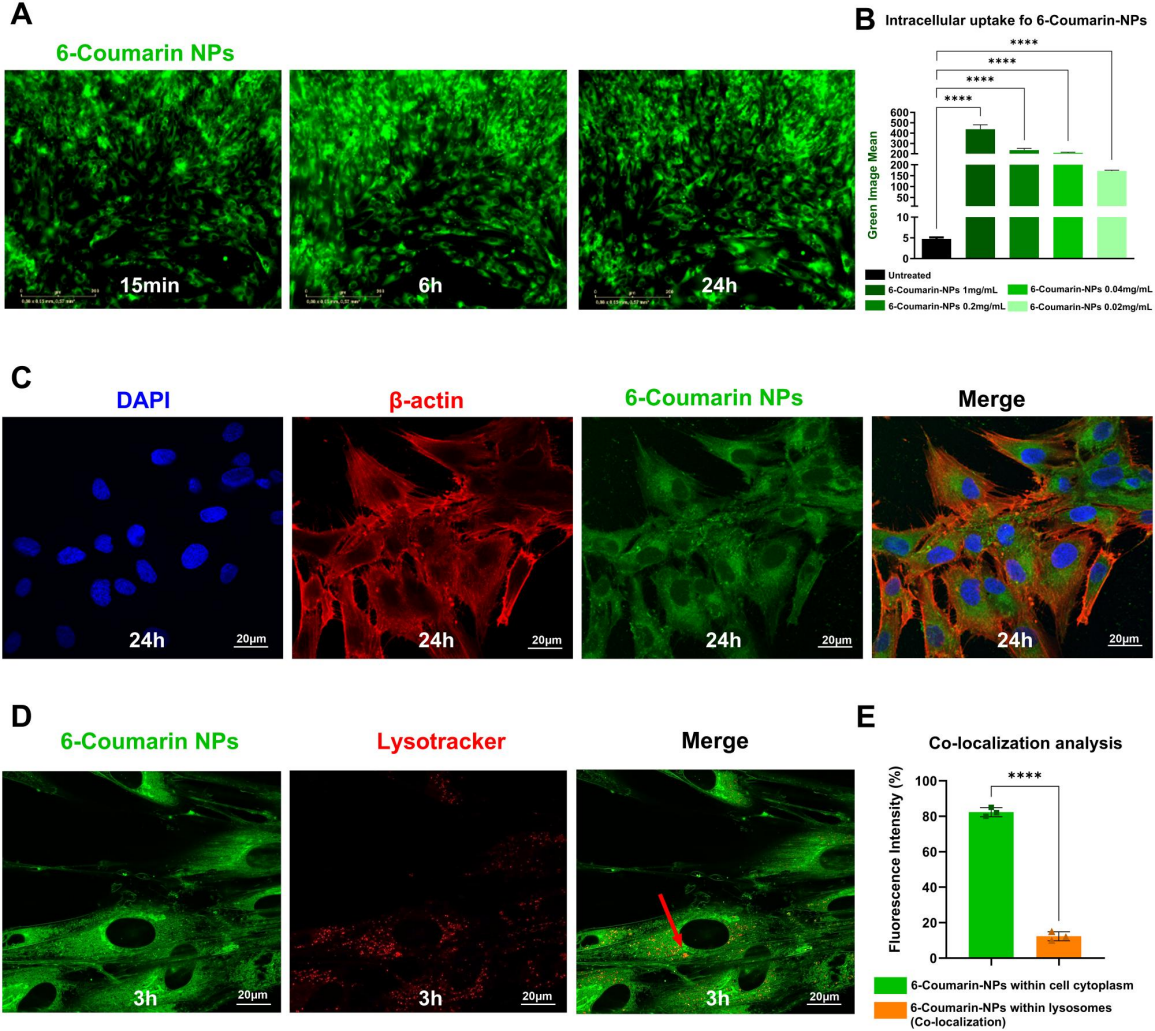
PLGA NP trafficking analysis. (**A**) Representative images detected by Incucyte Live-cell analysis system (10×) of CMSCs incubated with 0.02 mg/mL 6-Coumarin-NPs for 15 min, 6 h and 24 h. (**B**) Fluorescence quantification analysis of 6-Coumarin-NPs taken up by CMSCs after 24 h of incubation with scalar NP concentrations (from 1 to 0.02 mg/mL). Fluorescence signals were quantified in terms of green objects/total image area. Data were analysed using the one-way ANOVA test. ****P ≤ 0.0001. (**C**) Representative confocal microscopy images (60×) after 24 h of CMSCs incubation with 0.02 mg/mL 6-Coumarin-NPs. DAPI and β-actin staining were used to detect nuclei (in blue) and cytoskeleton (in red), respectively. (**D**) Representative confocal microscopy images (100×) of cells treated with Lysotracker Deep Red and incubated with 0.02 mg/mL 6-Coumarin-NPs for 3 h. Co-localization of 6-Coumarin-NPs and lysosomes is indicated by the red arrow in the merge image on the right. (**E**) Quantification of lysosomal escape based on co-localization analysis of 6-Coumarin-NPs and Lysotracker Deep Red staining using ImageJ software. Green bar: percentage of fluorescence derived from 6-Coumarin-NPs not overlapping with lysosomal signal (cytoplasmic distribution). Orange bar: percentage of co-localized fluorescence (NPs within lysosomes). Statistical analysis was performed using the unpaired Student’s *t*-test. ****P ≤ 0.0001. All experiments were performed in biological triplicate (*n* = 3). Results are shown as mean with SD (error bars).

The trafficking dynamics of 6-Coumarin-NPs were investigated through live-cell imaging using the Incucyte Live Cell Analysis System in NCS CMSCs. The live fluorescence imaging revealed a progressive increase in green fluorescence signal over time, starting as early as 15 min and increasing up to 24 h of incubation with NPs across all tested concentrations (Figure 6A and B). Fluorescence analysis demonstrated a concentration-dependent increase in the number of green fluorescent 6-Coumarin-NP spots (Figure 6B). Specifically, this analysis revealed an average of 172 green objects/image at the lowest concentration of NPs (0.02 mg/mL), increasing to over 440 green objects/image at the highest tested concentration (1 mg/mL) (Figure 6B).

A detailed investigation of NP biodistribution by confocal microscopy revealed that nanoparticles were broadly distributed throughout the cytoplasm of CMSCs, with no detectable signal in the nucleus and only partial co-localization with lysosomes (Figure 6C). To quantify lysosomal escape, fluorescence co-localization analysis was performed on confocal images of CMSCs treated with 6-Coumarin-NPs and labelled with Lysotracker. Our results showed that approximately 88% of 6-Coumarin fluorescence did not co-localize with lysosomal signal, indicating cytoplasmic distribution, whereas 12% of the signal was found within lysosomes (Figure 6E), highlighting only partial retention within the endo-lysosomal compartment.

To complement these observations, we extended the analysis to include the formulation based on low-Mw PLGA. For this purpose, additional high-magnification confocal images (100× objective) were acquired. These images, including orthogonal (z-stack) sections, confirmed the presence of fluorescent NPs within the cytoplasm of CMSCs, specifically in the same focal plane as the cell body and nuclei, thus excluding potential signal artefacts from out-of-focus planes (Supplementary Figure S3A and B). Additional images of low-Mw formulation, including lysosomal staining, were acquired at high magnification (100× objective with 2× digital zoom) to visualize potential NP accumulation within the endo-lysosomal compartment. As previously observed with the original formulation, NPs were widely distributed throughout CMSC cytoplasm with minimal overlap with lysosomes (Supplementary Figure S3C).

Overall, these analyses highlighted that PLGA NPs were suitable nanocarriers for intracellular delivery of therapeutic small molecules acting in the cytoplasmic compartment, such as siRNAs.

### PLGA NPs efficiently deliver siRNAs within the CMSCs’ cytoplasm compartment

**Figure 7:**
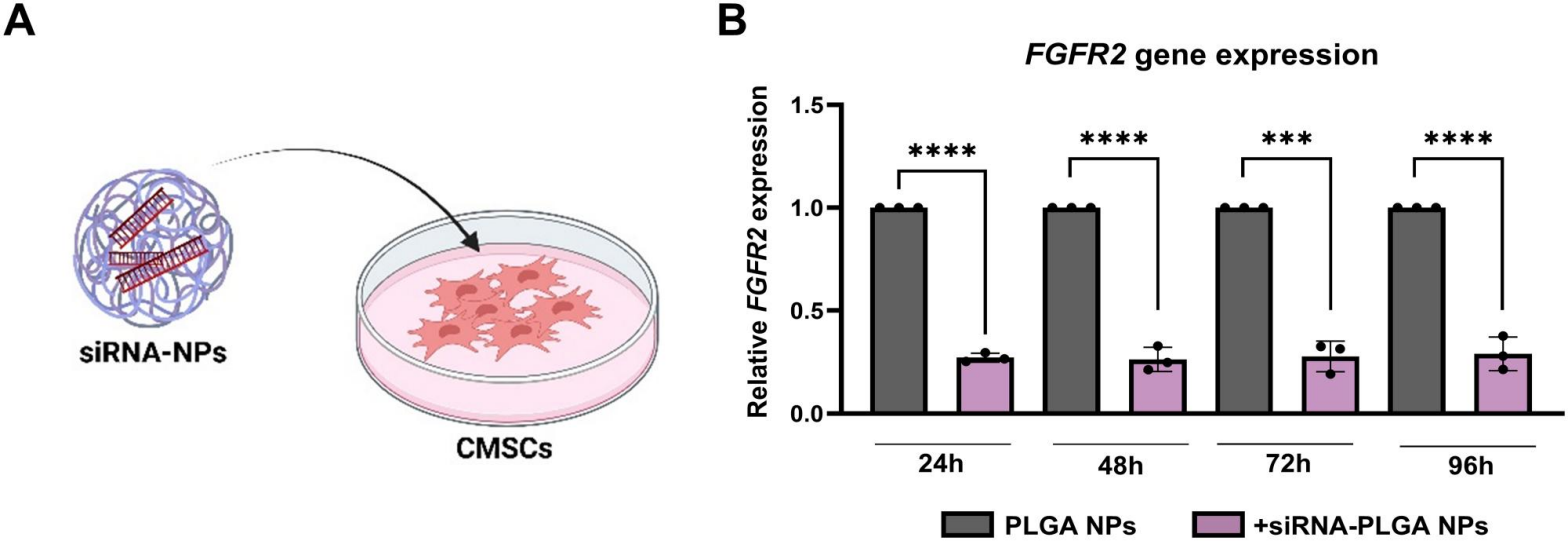
*FGFR2* Expression analysis after CMSC treatment with siRNA-NPs. (**A**) Schematic representation of CMSC treatment with siRNA-NPs. (**B**) *FGFR2* gene expression analysis in NCS-patient-derived CMSCs treated with 0.8 µM *FGFR2* siRNA-NPs complexes for 24, 48, 72 and 96 h by qPCR. Cells treated with empty-NPs were used as controls. Each condition was conducted in triplicate (*n* = 3). Results are shown as mean with SD (error bars). Data were analysed using the one-way ANOVA. ***P ≤ 0.001, ****P ≤ 0.0001.

The gene-silencing effect of siRNA-NP complex in patient-derived cells was assessed by analysing *FGFR2* expression levels at 24, 48, 72, and 96 h of treatment. Our results revealed a prolonged and stable mean inhibition of *FGFR2* transcript levels by 70% from 24 h up to 96 h of siRNA treatment (Figure 7B). Specifically, siRNAs led to 72% *FGFR2* downregulation in treated cells after 24 h, 74% after 48 h, 75% at 72 h and 71% after 96 h (Figure 7B). These data indicated that PLGA NPs were suitable for intracellular siRNA delivery, releasing these within cells cytoplasm and maintaining the target RNA-interfering effect over time.

Although a concentration of 10 nM was previously identified as the minimal effective dose of free siRNA to induce significant *FGFR2* knockdown in preliminary transfection experiments, a higher nominal concentration (0.8 µM) was used when loading siRNAs within the NPs. This adjustment was necessary to compensate for the encapsulation and release dynamics of the PLGA matrix, the gradual cytoplasmic availability of siRNA, and potential losses during intracellular trafficking. Therefore, the increased siRNA input ensured effective delivery to the target site and a prolonged RNA interference effect.

### GelXA ink can functionally release siRNA-NPs to surrounding cells

**Figure 8:**
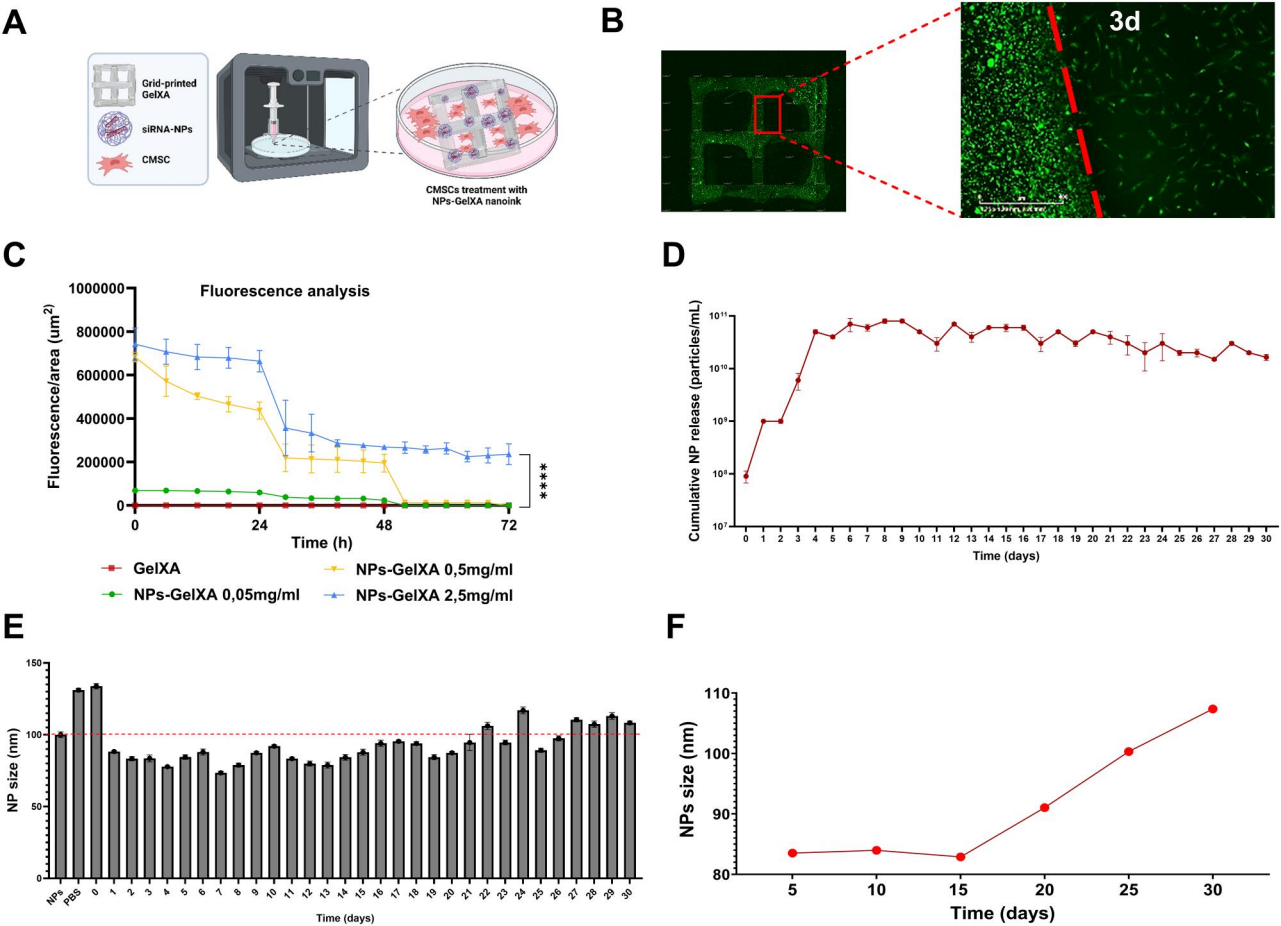
Release dynamics and stability of PLGA NPs from bioactive ink. (**A**) Schematic representation of 3D printing of the nanocomposite bioactive ink. (**B**) Representative images of trafficking analysis of 6-Coumarin-NPs (2.5 mg/mL) 3D-printed within GelXA hydrogel by Incucyte (10×). Dashed line: boundary between the ink and CMSCs seeded above. 3d: 3 days. (**C**) Fluorescence analysis of kinetic release of 6-Coumarin-NPs from the ink by Incucyte basic analyser. Statistical analysis was performed using the one-way ANOVA. ****P ≤ 0.0001. (**D**) Cumulative NP *in vitro* release profile of PLGA from GelXA ink up to 30 days in PBS at 37°C. (**E** and **F**) The bar graph (**E**) shows the nanoparticle (NP) size distribution measured by NTA over the 30-day release period, with error bars indicating standard error. NP size prior to encapsulation within the GelXA matrix is also shown (labelled as ‘NPs’) for comparison. ‘PBS’ refers to the size distribution of the blank control sample. The graph (**F**) displays the average NP diameter grouped into specific time intervals: days 0–5, 6–10, 11–15, 16–20, 21–25 and 26–30.

To enable a suitable platform for localized, controlled, and sustained delivery of PLGA NPs, we developed a mouldable system based on a 3D-printed nanocomposite bioactive ink composed of GelXA hydrogel embedded with PLGA NPs (Figure 8A). To evaluate this platform, CMSCs were incubated on the construct for 3 days, and NP release and cellular uptake were assessed in real time by tracking the green fluorescence signal of 6-Coumarin-NPs. As shown in Figure 8B, NPs were gradually released from the GelXA matrix into the culture medium (separated by the dashed red line in Figure 8B), and subsequently internalized by CMSCs located on adjacent surfaces. Fluorescence quantification confirmed rapid intracellular NP uptake within minutes of incubation, with the 2.5 mg/mL NP condition maintaining stable fluorescence levels over time (Figure 8C). Given the greater morphological homogeneity of low-Mw PLGA, we chose to perform long-term stability and release analyses using these NPs incorporated into the GelXA matrix. Long-term release analysis demonstrated a progressive increase in NP release with a rising trend from day 2 to day 4, which then stabilized and remained consistent over 30 days (Figure 8D). Particle size analysis revealed that smaller PLGA were released earlier, while larger particles were gradually released from day 15 onward (Figure 8E and F). Specifically, the mean diameter of the released NPs was 83.52 nm during the first 5 days and remained relatively stable up to day 15 (Figure 8F). From day 16, a progressive increase in size was observed, with the mean diameter reaching 91.04 nm between days 16–20, 100.32 nm during days 21–25, and up to 107.36 nm between days 26–30 (Figure 8F). These values are consistent with the average NP size distribution measured by NTA (∼100 nm) prior to incorporation into the GelXA matrix (Figure 8E). Together, these data validate the sustained and size-selective release of PLGA nanoparticles, supporting the potential of the nanocomposite system to achieve prolonged therapeutic effects through controlled delivery of bioactive molecules.

**Figure 9:**
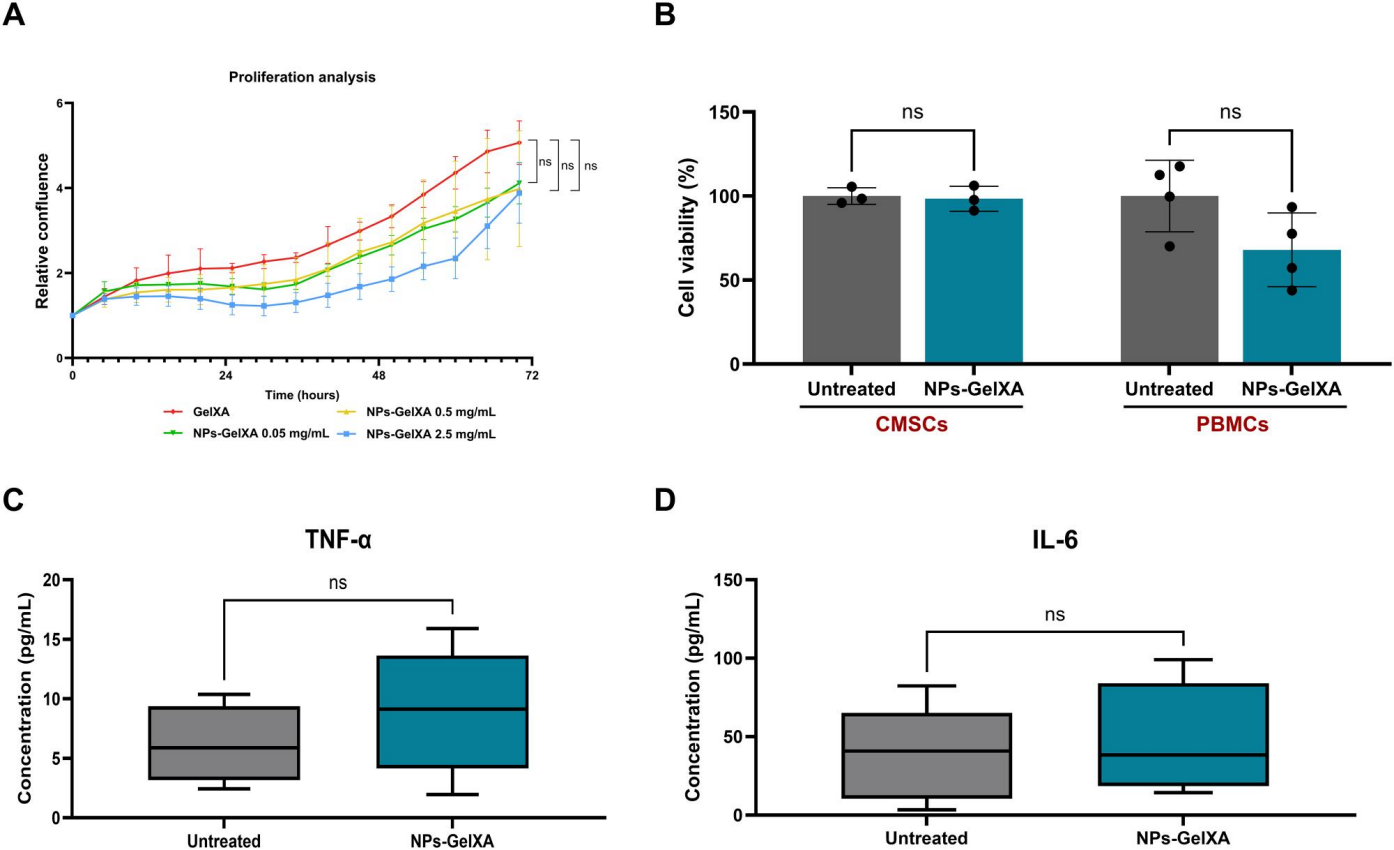
Cytocompatibility and immunogenicity of 3D-printed NP–GelXA constructs. (**A**) Proliferation analysis of CMSCs cultured with 3D-printed GelXA hydrogel matrix endowed with different concentrations of PLGA NPs (2.5, 0.5, 0.05 mg/mL) by Incucyte system. Statistical analysis was conducted using one-way ANOVA. (**B**) Graph showing the viability (%) of CMSCs and PBMCs incubated with the NP–GelXA system compared to untreated controls. Cytotoxicity data were normalized to their respective untreated controls. Results are shown as mean (*n* = 3 for CMSC and *n* = 4 for PBMCs) with SD (error bars). Statistical analysis was conducted using one-way ANOVA. (**C** and **D**) Quantification of pro-inflammatory cytokines TNF-α (**C**) and IL-6 (**D**) released by PBMCs derived from healthy donors following 24-h incubation with the NP–GelXA complex. Untreated PBMCs served as negative controls. Results were using Student’s *t*-test and are presented as box-and-whisker plots, showing the median, interquartile range, and minimum/maximum values. Each experiment was performed in biological quadruplicate (*n* = 4) and technical duplicate (*N* = 2). ns = not significant.

Furthermore, cytotoxicity analysis demonstrated that NP–GelXA treatment did not affect CMSC proliferation, as indicated by comparable confluence rates between treated and untreated cells over a 3-day incubation period across all tested conditions (Figure 9A). Similarly, CMSC viability remained unchanged following NP–GelXA exposure compared to controls (Figure 9B).

To evaluate the immunogenicity of the developed nanocomposite bioactive ink, we assessed PBMC viability and cytokine release profiles following incubation with the platform. As shown in Figure 9B, although a slight reduction in PBMC viability was observed, it was not statistically significant compared to untreated controls. Additionally, the quantification of pro-inflammatory cytokines, including TNF-α and IL-6, revealed comparable levels between PBMC incubated for 24 h with the NPs-GelXA system and untreated samples (Figure 9C and D). These data were further supported by the analysis of additional inflammatory cytokines such as CLXCL10; IL-1b; IL-13; IL-4 (Supplementary Figure S4A–D). Conversely, the levels of anti-inflammatory cytokines (e.g. IL1-RA) were slightly upregulated in the GelXA-treated group, although this increase was not statistically significant (Supplementary Figure S4F). Overall, these results supported the biocompatibility and low immunogenicity of the full nanocomposite hydrogel system.

**Figure 10:**
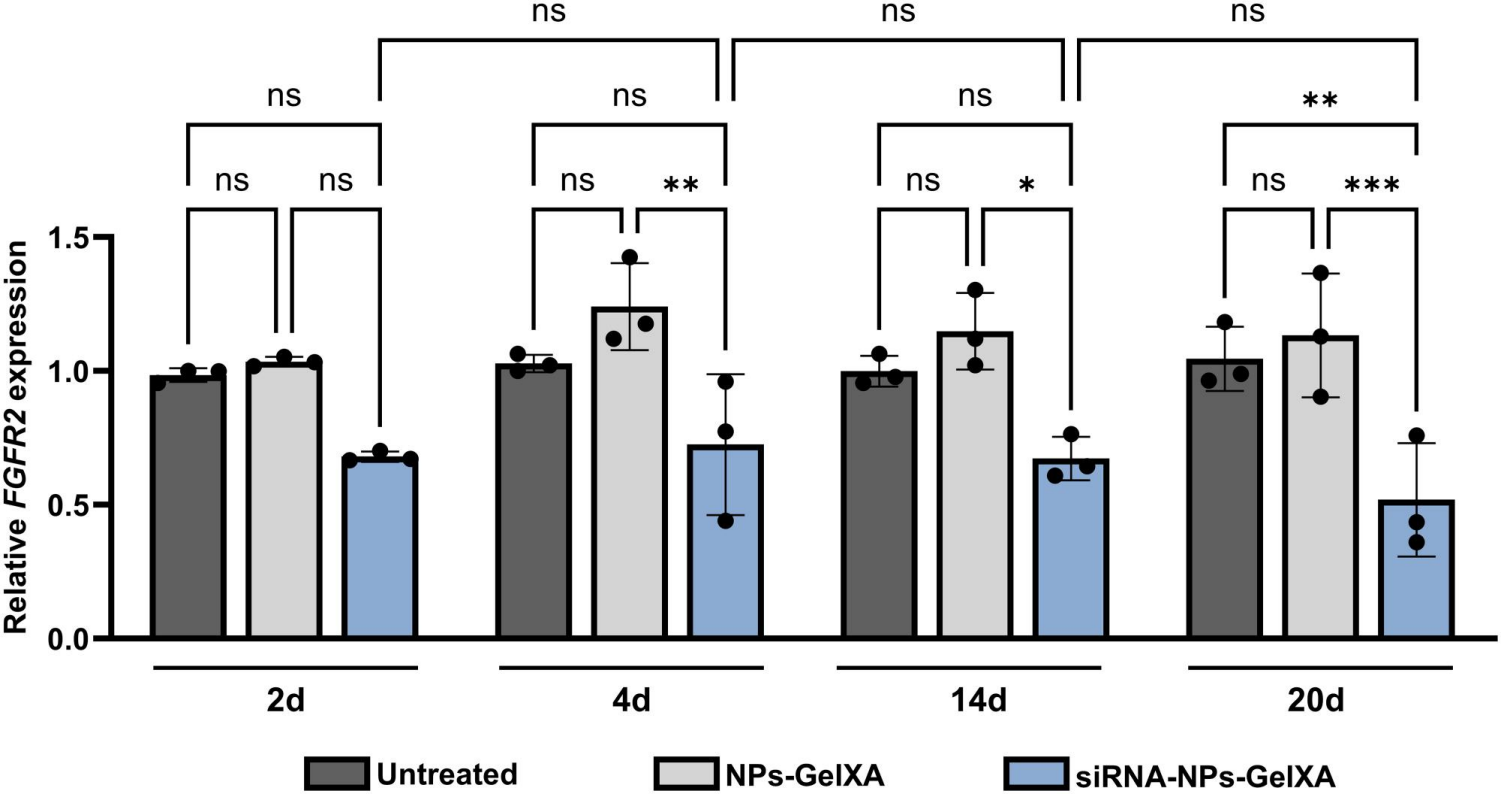
Time-course analysis of siRNA–NP–GelXA ink-mediated effect on target FGFR2 gene. *FGFR2* expression analysis of CMSCs treated with siRNA–NP–GelXA ink for 2, 4, 14 and 20 days. Untreated CMSCs and CMSCs seeded on GelXA containing empty-NPs (NP–GelXA) were used as controls. Results are shown as mean (*n* = 3) with SD (error bars). Statistical analysis was performed using one-way ANOVA. *P ≤ 0.05, **P ≤ 0.01, ***P ≤ 0.001; ns = not significant.

Finally, we tested the functional efficacy of siRNA-NPs released from the GelXA-based ink to assess their suitability for *in situ* delivery while preserving gene-silencing activity. Treatment with the siRNA-NP–GelXA ink, corresponding to a final siRNA concentration of 0.8 µM, resulted in a 30% downregulation of *FGFR2* after 2 days, compared to untreated controls (untreated CMSCs and CMSCs seeded on GelXA with empty-NPs) (Figure 10). Notably, this knockdown effect was sustained over time, with a consistent 30% reduction still observed at day 4, and further enhanced at later time points—reaching 33% at day 14 and up to 50% *FGFR2* downregulation after 20 days of treatment (Figure 10). No significant differences were observed between untreated CMSCs and those exposed to the delivery platform alone (GelXA combined with empty-NPs), confirming the specificity and safety of the system.

Overall, these results highlighted the prolonged and persistent gene-silencing effect achieved through the siRNA–NP–GelXA delivery system.

## Discussion

Although the only available treatment for CS relies on surgical procedures, the increased awareness of the molecular mechanisms involved in the etiopathogenesis of these defects has led to the identification of potential targets for the development of innovative therapeutic approaches [1, 23].

Therefore, a strategy that simultaneously targets dysregulated ossification signalling and supports *in situ* cranial defect regeneration could emerge as a valuable adjuvant approach to surgical treatment, offering the potential to enhance the currently challenging therapeutic outcomes for these patients.

In this context, the proposed therapeutic strategy is intended to develop a nanocomposite bioactive ink as *in situ* drug delivery system for hampering the aberrant FGFR2-mediated signalling occurring in CS patients’ calvarial MSCs, to modulate their abnormal osteogenic differentiation, hence allow for physiologic elasticity and critical expansion of the paediatric skull. The proposed bioactive nanocomposite could also contribute to overcoming the limitations of current bone grafting techniques by acting as a mouldable filler to stabilize and aid graft implant osteointegration.

In the craniofacial region, the FGFR2 receptor is predominantly expressed in osteoprogenitor cells within cranial suture mesenchyme and in differentiating osteoblasts at the sutural osteogenic fronts of the calvaria bones, and its signalling plays a critical role in skeletal development and morphogenesis [84, 87, 88]. Indeed, mutations involving *FGFR2* gene are responsible for the aberrant osteogenic commitment of the MSCs inhabiting the sutures, leading to their premature ossification [1, 23, 28, 89, 90].

Since the disease pathophysiology of both syndromic and NCS is largely attributable to the overactivation of FGF/FGFR signalling, in recent decades, several studies have demonstrated the feasibility of pharmacological agents targeting FGFR2 and its downstream mediators such as MAPK/ERK in preventing aberrant skeletal phenotypes in mouse models of CS [27, 91, 92]. The RNA interference strategy has shown promise in reprogramming cells in mouse models of CS [26, 27, 28]. Specifically, Shukla and colleagues demonstrated that a small hairpin RNA (shRNA) targeting the dominant mutant allele of *Fgfr2* (*Fgfr2^S252W^*) completely prevented Apert syndrome phenotype in mice through inhibition of the ERK1/ERK2 pathway cascade and downstream target genes [27]. Moreover, Luo and collaborators developed allele-specific siRNA complementary to the mutant *Fgfr2* allele carrying Pro253Arg(P253R) mutation capable of discriminating and inhibiting the mutated allele. Following treatment with adeno-associated virus 9 (AAV9)-based vector delivering siRNA in the cranial sutures of Apert syndrome (*Fgfr2^+/P253R^*) mouse, they observed a reduction in the rate of premature coronal suture fusion and a decrease in skull bone density [26].

In this context, we recently designed customized sequence-specific siRNAs targeting the mutant *FGFR2* allele, effectively restoring the overactive FGFR2 signalling cascade and correcting the aberrant osteogenic commitment of cells isolated from calvaria suture tissues of Crouzon syndrome patients with different single nucleotide substitutions in the *FGFR2* gene [28].

In this study, we first assessed the central role of FGFR2 dysregulation in CS by demonstrating altered basal FGFR2 signalling in CMSCs derived from pathological sutures of CS patients. Notably, this overactivation was also observed in NCS patients without any known *FGFR2* pathogenic mutations, suggesting that aberrant FGFR2 signalling contributes to disease pathophysiology even in the absence of identified genetic alterations, as previously described in syndromic forms such as Crouzon syndrome [93, 94] To counteract this abnormal activity, we selected a pool of siRNAs specifically targeting the *FGFR2* mRNA, which exhibited high target gene specificity, supporting a stable and sustained knockdown effect—without inducing compensatory overexpression or off-target effects upon treatment. CMSC treatment with selected siRNAs resulted in efficient *FGFR2* silencing of both mRNA (70–90%) and protein levels (20–40%). The observed difference in knockdown efficiency aligns with the known mechanism of siRNAs, which predominantly degrade target mRNA, whereas protein reduction is also influenced by factors such as half-life, cellular turnover, and post-transcriptional regulation. Notably, FGFR2, like other receptor tyrosine kinases, may have a relatively long half-life, potentially attenuating the reduction in protein abundance within the experimental timeframe.

Functionally, siRNA-mediated FGFR2 reduction also attenuated FGFR2 aberrant activation and downstream signalling, as evidenced by decreased phosphorylation of FGFR2 and of its downstream effectors, including ERK1/2 and the osteogenic transcription factor RUNX2.

Mechanistically, the activation of FGFR2 promotes receptor dimerization and its trans-self-phosphorylation [95, 96]. This event triggers downstream signalling, including activation of the RAS–RAF–MEK–ERK cascade [97]. Activated ERK1/2 phosphorylates among their targets, the master bone transcription factor RUNX2, enhancing its stability and transcriptional activity, thereby promoting the expression of osteogenic genes essential for cranial suture ossification [98]. Accordingly, our data demonstrated that FGFR2 silencing impaired ERK1/2 phosphorylation and reduced RUNX2 expression, thereby validating the use of FGFR2-targeting siRNAs as a promising strategy to modulate pathological ossification in cranial sutures [96, 99].

Moreover, the therapeutic potential of targeting FGFR2 to slow down the aberrant osteogenic behaviour of CMSCs through selected siRNA molecules was also confirmed in *FGFR2*-mutation-positive patients with syndromic CS form such as those with Crouzon syndrome, whose pathophysiology is directly linked to mutations in the *FGFR2* gene. In the cells from these patients, siRNA treatment produced a similar attenuation of FGFR2-associated signalling as observed in NCS cases. These data supported that RNA interference mediated by siRNAs is a suitable therapeutic approach to restore the physiological signalling in CMSCs for patients suffering from CS, including those with more severe and complex syndromic cases. However, siRNA-based therapies are hindered by limitations related to their low chemical stability, the easy degradation by nucleases and lysosomes, the possible activation of the immune system, the off-target effects, and their negative charge and hydrophilicity that make them poorly permeable to cross the phospholipid bilayers of cell membranes [29, 30, 31]. To overcome these limitations, increase siRNAs biodistribution and improve their pharmacokinetic profile, we developed biocompatible nanocarriers based on PLGA NPs able to entrap siRNAs within their core [68, 100, 101]. The produced PLGA NPs proved to be biocompatible in our cellular model, where they did not affect cell viability and proliferation. Our data also highlighted that the PLGA NPs were efficiently and rapidly uptaken by CMSCs where they were retained in the cytoplasm with only partial and negligible co-localization within lysosomes. This observation suggested that our NPs can release siRNAs into the cytoplasm, where they are directed by the RNA-induced silencing complex to the target mRNA for degradation. To validate this, NPs loaded with siRNAs targeting the *FGFR2* gene were produced and tested in patients’ cells. To improve the upload of nucleic acids into the negatively charged PLGA NPs, siRNAs were conjugated with poly-L-lysine. The complexation with a cationic agent like poly-lysine is also exploited to improve the stability of siRNA, to enhance cytoplasmic localization and favour the endosomal escape through the “proton sponge effect” [82, 100]. The NPs efficiently encapsulated *FGFR2* siRNAs, and once internalized by cells, the siRNA-NP complexes successfully escaped the endo-lysosomal trafficking route. This allowed for the release of siRNAs into cytoplasmic compartments, preserving their biological activity and achieving a stable, prolonged *FGFR2* knockdown in patients’ cells. Collectively, these results validated the suitability of the PLGA-based nanodelivery system for the intracellular release of functionally effective siRNAs.

To achieve site-specific release of bioactive NPs and ensure sustained, time-controlled RNA interference, we developed a functionalized nanocomposite ink by incorporating siRNA-NPs into a GelMA-based hydrogel, widely used in biomedical applications for its biocompatibility and tunable properties, particularly in the context of bone tissue engineering. In fact, GelMA functionalized with allogenic exogenous MSCs has already been used to successfully restore cranial defect while preventing re-ossification of suture (re-synostosis) in a CS *Twist1*^+/−^ mice model [102]. Furthermore, an *in situ* injectable hydrogel was used in a CS mouse model for the controlled administration of Gremlin1, a BMP inhibitor. *In situ* application of this functionalized hydrogel demonstrated that Gremlin1 was able to inhibit the rapid bone growth of the pathological suture for up to 14 days post-surgery [11, 12]. Among the commercial inks used in tissue engineering, especially for cranio-maxillofacial regeneration, CELLINK’s GelXA, consisting of GelMA base with xanthan gum and alginate, has already been used in combination with MSCs to form a bio-ink for the repair of the cranial frontal bone [103, 104].

In this work, GelXA was used to produce the functionalized 3D-printed ink for delivering selected therapeutic siRNAs to modulate the aberrant intracellular signalling responsible for premature osteogenic differentiation of CMSCs in skull sutures, which drives CS.

Fluorescence analysis and release profile studies confirmed that the 3D-printed siRNA–NP–GelXA ink effectively retained the NPs within the hydrogel mesh during cell treatment. This retention enabled controlled and sustained release kinetics of morphologically intact and functionally stable NPs both into the surrounding medium and in the immediate cellular microenvironment. Furthermore, despite all tested NP preparations—including high- and low-Mw PLGA—exhibiting comparable biocompatibility and siRNA encapsulation yield, we observed that low-Mw PLGA NPs (30 kDa) displayed superior morphological homogeneity. This feature, considered critical for ensuring reproducibility and long-term structural stability, guided their selection for extended release and stability evaluations. Specifically, the smaller size of low-Mw PLGA NPs, combined with the cross-linking density of the GelMA matrix, enabled a gradual and prolonged NP release from the nanocomposite ink over 30 days. Notably, particles with smaller diameters were released earlier, followed by those with larger sizes—highlighting the influence of NP size and matrix architecture on the release profile, as previously reported in other tissue engineering applications [105]. To support the further clinical translatability of this approach, we have also evaluated the immunological response of PBMC by studying the release profile of key pro- and anti-inflammatory cytokines following exposure to NP–GelXA ink. The results showed no significant upregulation of TNF-α, IL-6, or IL-1β, nor consistent alterations in IL-10 and IL-4 levels, suggesting that the NP–GelXA formulation does not elicit an overt immune response *in vitro*. These data supported the biocompatibility and immunological safety of the delivery system, which are essential criteria for future therapeutic application, particularly in paediatric settings.

In addition, we demonstrated that the NPs released by the NP–GelXA ink were efficiently endocytosed by CMSCs cultured in the hydrogel adjacent area, causing a significant and stable inhibition of the *FGFR2* target gene in cells for up to 20 days of treatment. This suggests that GelXA did not interfere with either the NPs-mediated siRNA delivery or the biological activity of the therapeutic siRNA, but rather enhanced its sustained release and provided physical support.

Taken together, our results suggest that the developed nanocomposite ink represents a promising delivery strategy to modulate pathological signalling in cranial sutures of CS patients. By supporting both therapeutic efficacy and immunological tolerability, this platform offers a solid foundation for advancing toward translational applications in tissue engineering and paediatric cranioplasty, while avoiding the drawbacks of systemic NPs administration.

## Conclusion

This study demonstrates the efficacy of a novel formulation of a GelMA-based ink enriched with PLGA NPs as a functionally effective delivery platform for RNA interference-based therapy for craniosynostoses (CS). The localized and sustained release of *FGFR2*-targeting siRNA from the hydrogel at the calvaria defect site could modulate the osteogenic phenotype of CMSCs, thereby hindering the aberrant and premature fusion of cranial sutures characteristic of CS. This technology offers a promising adjunct to current surgical strategies, particularly in patients for whom extensive cranial remodelling remains the gold standard.

Thanks to its mouldable and injectable properties, the nanocomposite bioactive ink could serve as a bioactive sealant—stabilizing implants, preventing graft dislocation, and enabling localized drug delivery *in situ*. Furthermore, the modular design of this system allows for adaptation to other therapeutic applications, including the delivery of antibiotics or other customized therapeutics.

Ongoing preclinical studies aim to evaluate the safety, mechanical performance, and therapeutic efficacy of this system *in vivo*, as a critical step toward clinical translation. Notably, the materials and technologies used in this platform are already under investigation in several clinical trials (ClinicalTrials.gov: NCT06533150, NCT06011551, NCT04377256, NCT06715345, NCT05898074, NCT03800797, NCT04727385, NCT01295060, NCT04840147, NCT04293861), potentially accelerating regulatory approval and facilitating rapid integration into clinical practice. Overall, this platform represents a significant advancement in craniofacial regenerative medicine, offering a minimally invasive, customizable therapeutic solution for complex paediatric skull defects.

## Supplementary Material

rbaf115_Supplementary_Data
