# Supplementary material for: A smart nanocomposite bioactive ink for controlled siRNA delivery in calvarial mesenchymal stromal cells as a minimally invasive treatment for craniosynostosis
Source: Regen Biomater. 2025 Nov 8;13:rbaf115. doi: 10.1093/rb/rbaf115 (PMC12869795; doi:10.1093/rb/rbaf115)
Supplement: rbaf115_Supplementary_Data [file rbaf115_supplementary_data.zip › 15-Nov-2025_013314_Edit_Supplementary_Figures_Salvati_et_al.,_2025.docx]

**Supplementary Figures:**

**Supplementary Figure 1. siRNA validation in Crouzon patients.** Gene expression analysis of Crouzon-patient derived CMSCs treated with 10 nM of a siRNA pool targeting *FGFR2* delivered by Lipofectamine for 48 h. Cell treated with Lipofectamine alone were used as vehicle controls (Lipofectamine). Results are shown as mean (n = 3) with SD (error bars). Statistical analysis was performed using Student’s t-test. *** *P* ≤ 0.001.

**Supplementary Figure 2. Characterization of PLGA NPs.** (**A-C**) Size distribution (diameter) by intensity (%) of empty NPs (in violet), 6-Coumarin-NPs (in red) and siRNA NPs (in yellow) and using dynamic light scattering (DLS). (**D**) Bar graph summarizing mean hydrodynamic diameters for each NP formulation as measured by DLS. (**E**) Z-potential values obtained for empty NPs (in pink) and siRNA NPs (in yellow). *P ≤ 0.05, **P ≤ 0.01, ***P ≤ 0.001.

**Supplementary Figure 3**: **Intracellular PLGA NP trafficking.** (**A**) Representative confocal microscopy images (100x) after 24 h of CMSCs incubation with 0.02 mg/mL 6-Coumarin-NPs. DAPI and β-actin staining were used to detected nuclei (in blue) and cytoskeleton (in red), respectively. Merged images include orthogonal (XZ and YZ) projections to better visualize the spatial distribution of NPs (green) within the cellular volume and confirm cytoplasmic localization. (**B**) 3D surface reconstruction of the image in panel A, generated from Z-stack acquisition, illustrating the three-dimensional distribution of NPs in relation to the actin cytoskeleton and nuclei. (**C**) Representative confocal images acquired at 100× with 2× digital magnification of cells treated with Lysotracker deep red and incubated with 0.02 mg/mL 6-Coumarin-NPs for 3 h.

**Supplementary Figure 4**: **Inflammatory response of NP-GelXA ink.** Quantification of pro-inflammatory cytokines CLXCL10 (**A**) IL-1b (**B**) IL-13 (**C**) and IL-4 (**D**) and anti-inflammatory cytokines IL-10 (**E**) IL1-RA (**F**) released by PBMCs derived from healthy donors following 24-hour incubation with the NP-GelXA complex. Untreated PBMCs served as negative controls. Results were analyzed using Student’s t-test and presented as box-and-whisker plots, showing the median, interquartile range, and minimum/maximum values. Each experiment was performed in biological quadruplicate (n = 4) and technical duplicate (N = 2). ns = not significant.
